# Real-time prediction of ^1^H and ^13^C chemical shifts with DFT accuracy using a 3D graph neural network†

**DOI:** 10.1039/d1sc03343c

**Published:** 2021-08-09

**Authors:** Yanfei Guan, S. V. Shree Sowndarya, Liliana C. Gallegos, Peter C. St. John, Robert S. Paton

**Affiliations:** Department of Chemistry, Colorado State University Fort Collins CO 80523 USA yanfei.guan@pfizer.com robert.paton@colostate.edu; Biosciences Center, National Renewable Energy Laboratory Golden CO 80401 USA

## Abstract

Nuclear magnetic resonance (NMR) is one of the primary techniques used to elucidate the chemical structure, bonding, stereochemistry, and conformation of organic compounds. The distinct chemical shifts in an NMR spectrum depend upon each atom's local chemical environment and are influenced by both through-bond and through-space interactions with other atoms and functional groups. The *in silico* prediction of NMR chemical shifts using quantum mechanical (QM) calculations is now commonplace in aiding organic structural assignment since spectra can be computed for several candidate structures and then compared with experimental values to find the best possible match. However, the computational demands of calculating multiple structural- and stereo-isomers, each of which may typically exist as an ensemble of rapidly-interconverting conformations, are expensive. Additionally, the QM predictions themselves may lack sufficient accuracy to identify a correct structure. In this work, we address both of these shortcomings by developing a rapid machine learning (ML) protocol to predict ^1^H and ^13^C chemical shifts through an efficient graph neural network (GNN) using 3D structures as input. Transfer learning with experimental data is used to improve the final prediction accuracy of a model trained using QM calculations. When tested on the CHESHIRE dataset, the proposed model predicts observed ^13^C chemical shifts with comparable accuracy to the best-performing DFT functionals (1.5 ppm) in around 1/6000 of the CPU time. An automated prediction webserver and graphical interface are accessible online at http://nova.chem.colostate.edu/cascade/. We further demonstrate the model in three applications: first, we use the model to decide the correct organic structure from candidates through experimental spectra, including complex stereoisomers; second, we automatically detect and revise incorrect chemical shift assignments in a popular NMR database, the NMRShiftDB; and third, we use NMR chemical shifts as descriptors for determination of the sites of electrophilic aromatic substitution.

## Abstract 2

From quantum chemical and experimental NMR data, a 3D graph neural network, CASCADE, has been developed to predict carbon and proton chemical shifts. Stereoisomers and conformers of organic molecules can be correctly distinguished.

## Graphical abstract

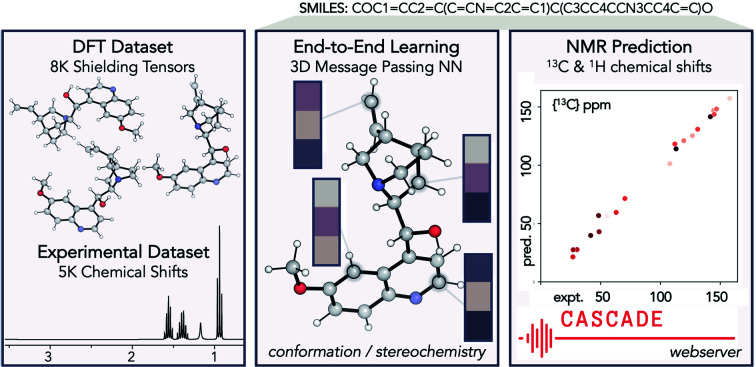

## Introduction

Nuclear magnetic resonance (NMR) spectra are a primary source of molecular structural information. NMR chemical shifts report detailed information on atoms' local chemical environments that can be used to determine the atomic connectivity, relative stereochemistry and conformations of molecules. Organic structure assignment has for many years been performed manually, however, recent advances in computational chemistry have paved the way for the *in silico* prediction of chemical shifts. Comparisons of experimental *isotropic* chemical shifts (*i.e.*, those measured for solution samples) with computationally predicted values have been applied, sometimes including scalar coupling constants, to various problems in structure elucidation: the assignment of relative stereochemistry in flexible organic molecules as pioneered by Bagno and Bifulco,^1–3^ complex natural product structure elucidation and reassignment,^4–6^ identification of the side product(s) in synthetic reactions,^7,8^ deducing the macromolecular conformation adopted by cyclic peptides,^9^ and in correcting literature misassignments.^10^ The growing importance of computational chemical shift prediction, particularly of ^13^C and ^1^H nuclei, in natural product, mechanistic and synthetic organic chemistry is the subject of an authoritative review by Tantillo and co-workers.^11^

To serve as a useful tool for structure elucidation, prediction errors in computed chemical shifts must be smaller than the experimental variations between different candidate structures. To this end, empirical correction schemes for density functional theory (DFT) computed shielding tensors have been instrumental in improving the levels of accuracy: Tantillo and co-workers^11^ derived and compiled linear-scaling parameters for many levels of theory, basis set and solvation models (in the CHESHIRE repository^12^), and have established standardized molecular training and test sets for chemical shift prediction. Alternative correction schemes to improve computational results have been developed using multiple external standards^13,14^ and atom-based correction factors.^15,16^ As a result, contemporary “best practice” DFT protocols boosted by empirical corrections routinely approach accuracies of 2.5 ppm in the prediction of ^13^C shifts, or 0.15 ppm for ^1^H shifts, expressed as root mean square error (RMSD).^11^ The quantitative application of these predictions to organic structure elucidation has been pioneered by Goodman and co-workers^17,18^ in the development of CP3, DP4 and DP4+ parameters,^19^ the latter of which provides a statistical estimate for the confidence of a particular computational structural assignment. Ermanis and Goodman recently introduced the DP4-AI platform, which enables automated stereoisomer elucidation directly from a ^1^H and ^13^C spectrum.^20^ In general, however, the time and computational resources associated with quantum chemical approaches can be significant, particularly for large and conformationally flexible molecules.^21^ Even with access to high-performance computing resources, the consideration of multiple structures in a high-throughput manner is highly challenging at present.

Empirical approaches to chemical shift prediction provide a less expensive alternative to electronic structure calculations by harnessing pre-existing knowledge such as large datasets of experimentally measured chemical shifts. Additive methods have been developed to predict chemical shift based on the cumulative effects of local substituents, as implemented in *ChemDraw*.^22^ More sophisticated machine learning (ML) methods encode each atom as a one-dimensional vector using an atom-based connectivity scheme. For example, a hierarchically ordered spherical description of environment (HOSE) code^23^ predicts chemical shifts based on the measured similarity to database entries or by using fully-connected neural networks.^24–29^ When trained against a large number of experimentally measured chemical shifts, these methods have achieved predictive accuracies of 1.7 ppm for ^13^C chemical shifts and 0.2 ppm for ^1^H shifts (expressed as mean absolute error, MAE).^24^ These earlier ML approaches tend to rely upon *feature engineering*:^30^ expert-crafted rules are required to encode atomic environment, which can suffer from human bias and incompleteness, and which are often trained separately for different atom types (*e.g.*, different models are developed for tetrahedral and trigonal carbon atoms). In particular, the rise of *feature learning*, as embodied by graph neural networks (GNNs),^31^ has enabled ‘end-to-end’ learning from molecular structures and avoids rule-based encoding. Jonas and Kuhn^32^ have developed a GNN to predict the ^13^C and ^1^H chemical shifts and achieved an accuracy of 1.43 ppm for ^13^C and 0.28 ppm for ^1^H (MAE for the testing set) using 2D molecular connectivity as input. Recently a GNN architecture was described that can capture the effect of noncovalent interactions and secondary structure effects on chemical shifts of C, N and H nuclei in biomacromolecules and organic molecules.^33^

**Scheme 1:**
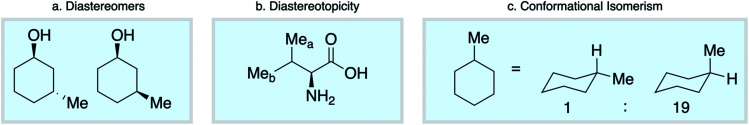
Stereochemical and conformational influences on chemical shift.

Empirical approaches to NMR chemical shift prediction use interatomic connectivity to define the local neighborhood around a given atom, while the effects of stereochemistry and molecular conformation are most often ignored. However, geometric factors play a fundamental role in influencing chemical shift. Diastereoisomers of a given compound are distinguishable by NMR (Scheme 1a), as are diastereotopic atoms or groups within the same molecule (Scheme 1b). Furthermore, molecular conformations give rise to different chemical shifts that may appear as distinct signals or as ensemble-averaged values depending on the interconversion rate relative to the NMR timescale (Scheme 1c). Such phenomena are not conveniently captured by the commonly-used descriptions of atomic environments that only encode local connectivity. Although DFT chemical shift predictions are now routinely used to differentiate stereoisomers, empirical approaches based on the 2D molecular graph fail this task absolutely. We reasoned that this challenge could be directly addressed by a model that uses a spatial representation of atomic environments in the form of a 3D molecular graph.^34^ Interatomic distances, including both bonded and nonbonded interactions, are an inherent part of this description, which is therefore able to capture variations in chemical shift across diastereoisomeric molecules, diastereotopic groups within a single chiral molecule, and spatially distinct molecular conformations.

Unlike the valence bond model of chemical structure, 3D representations of local atomic environments such as atom-centered symmetry functions,^35,36^ do not require pre-conceived rules concerning topology, chemical bonding, or other physicochemical descriptors. These and related representations have been widely applied to predict atomic and molecular properties by ML methods.^37–43^ We surmised that the prediction of NMR chemical shift, being strongly influenced by local environment and stereochemistry, would be amenable to such an approach, although this has received limited attention.^44,45^ Using a sorted Coulomb matrix^46^ to represent atomic environments, von Lilienfeld and co-workers^44^ have predicted shielding tensors for small organic molecules by kernel ridge regression (KRR),^47^ obtaining MAEs of 3.9 ppm for ^13^C and 0.28 ppm for ^1^H relative to DFT values. However, the moderate levels of accuracy and reliance on DFT optimized structures as inputs limit practical applications to chemical structure elucidation. Using a smooth overlap of atomic positions (SOAP) kernel^48^ to evaluate the correlation between local atomic environments, Ceriotti and co-workers^45^ performed Gaussian process regression in a seminal work^49^ to predict shielding tensors of molecular solids with RMSEs of 4.3 ppm for ^13^C and 0.49 ppm for ^1^H. Their model was able to assign the crystal polymorphic of cocaine from a selection of candidate structures by comparing against experimental chemical shifts. Another machine learning model, IMPRESSION, involving kernel ridge regression was developed by Butts and co-workers, where they leverage DFT-computed NMR parameters to predict ^1^*J*_CH_ scalar couplings and ^13^C and ^1^H chemical shifts with an MAE of 0.87 Hz, 0.23 ppm and 2.45 ppm respectively for an independent test set.^50^ Community-powered approach has also been sought to improve the prediction of NMR properties, where they develop a combined model which was 7–19 times more accurate than existing prediction models.^51^ Herein, we develop a GNN model to predict isotropic ^13^C and ^1^H chemical shifts from a 3D representation of atomic environments. The favorable levels of accuracy and speed permit structural and stereochemical assignments to be carried out for large and flexible organic molecules that would be enormously challenging for quantum chemical approaches.

## Approach

Empirical chemical shift prediction models require large amounts of experimental data. Although a large number of NMR spectra certainly exist, the majority of these are in a form not readily utilized by ML methods. NMR data and the assignment of experimental shifts to specific atoms in molecular structures are processed and reported in a variety of formats that are difficult to parse automatically.^52^ Additionally, the literature contains assignment errors, incompletely recorded spectral data, and partially assigned structures. Manually-curated datasets have thus featured heavily in the development of predictive models for chemical shifts,^24^ requiring considerable effort and expertise to build and maintain. The NMRShiftDB^53^ stands as an exception to this approach, being an open-submission and open-access database containing around 400 000 experimental ^13^C chemical shifts. However, the frequency of incorrect assignments has been debated in the literature,^26,29^ and incomplete annotation of stereochemistry affects a significant proportion of chiral molecules contained in this dataset. The need for a repository of publicly accessible raw NMR data has been articulated elsewhere.^54^

To address these challenges, we set out to exploit advances in quantum chemistry, high-performance computing, and automation in developing a large dataset of QM computed values to train an ML model.^38,40,44,45,55–57^ A principal advantage of this approach is that DFT-based predictions of chemical shifts can be mapped to the responsible atom in a high-throughput fashion with complete reliability, avoiding incomplete or erroneous assignments and the need for manual intervention. Datasets containing 100 000 ^13^C and ^1^H chemical shifts are readily attainable *via* automation (see below), and the conformational dependence of chemical shifts can be effectively learned by the inclusion of different molecular geometries. Without experimental data, however, the predictive accuracy of any prospective ML model is fundamentally limited by the underlying performance of the DFT methodology, basis set, description of solvation, and other sources of computational error. Therefore, we pursued a transfer learning (TL) strategy,^58,59^ inspired by the work of Roitberg, Isayev, and co-workers^60^ in which the accuracy of a NN potential extensively trained against DFT energetics could be enhanced using a much sparser dataset of high-quality CCSD(T) values. We demonstrate improvements in the predictive accuracy of a DFT-trained model by applying TL with a smaller collection of experimental values: following model retraining against a curated set of ^13^C experimental shifts, a mean absolute error (MAE) of 1.23 ppm against experiment could be obtained for 500 held-out structures (see below). This involved additional 5000 experimental structures to the existing 8000 DFT optimized structures. Taking a step further, we demonstrate that molecular geometries obtained from inexpensive molecular mechanics calculations can be used directly without a substantial loss in accuracy, generating chemical shift predictions on the order of 5–10 000 times faster than conventional electronic structure calculations.

## GNNs for atomic property prediction

**Fig. 1:**
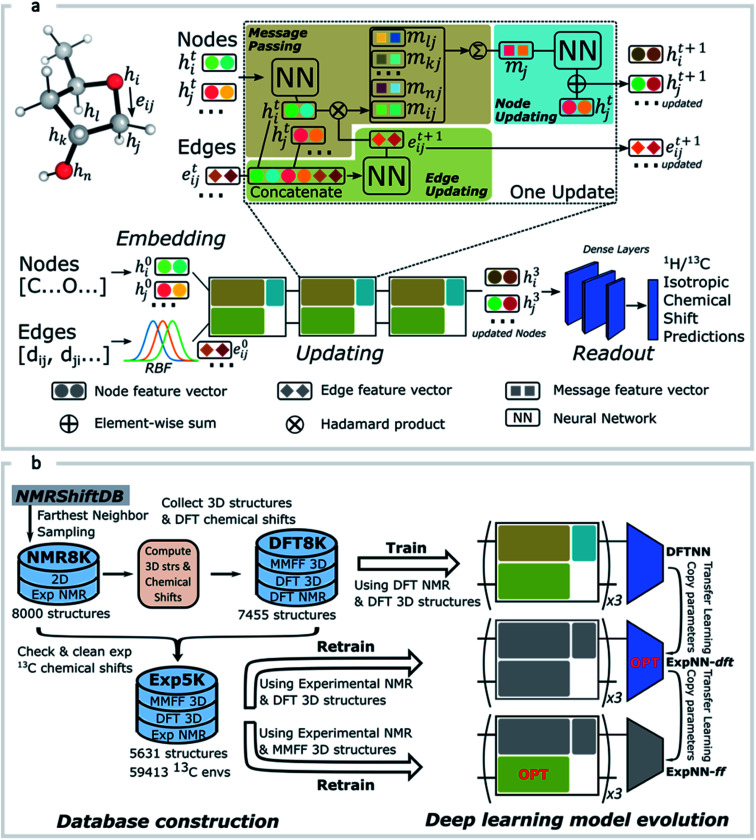
(a) Illustration of the GNN architecture. Molecules are represented according to their atom types and interatomic distances. Each atom, or node, is embedded as a vector of atomic attributes. Each atom pair within a distance of 5 Å is linked by an edge, which is embedded into a continuous vector with a set of radial basis functions (RBF). Node and edge feature vectors are then iteratively updated by the updating blocks, through which each atom is responsible for learning atomic features by message passing. Updated node features for all ^1^H or ^13^C atoms then pass through a series of dense layers to yield final chemical shift predictions. (b) Data processing workflow. NMR8K is a primary dataset composed of 8000 2D structures along with unchecked experimental chemical shifts sampled from NMRShiftDB directly; DFT8K is the corresponding dataset we generated by appending MMFF/DFT optimized 3D structures and GIAO chemical shifts; “Cleaned” experimental chemical shifts filtered by DFT results as well as corresponding 3D structures are stored in Exp5K. Three distinct GNN models were trained on these datasets. During transfer-learning, we fixed a subset of network parameters, shaded in grey, while the OPT block indicates optimizable parameters. Model ExpNN-ff, trained against DFT and experimental chemical shifts while processing molecular mechanics geometries as inputs, has been developed into a web-based predictor.

GNNs^31,55,61–69^ do not depend on pre-computed descriptors and are able to learn underlying regularities directly from the molecular graph, represented either in 2D form, encoding interatomic connectivity, or in 3D form, where spatial information is included. GNNs have recently been applied to end-to-end (*i.e.*, structure-to-property) learning of molecular properties such as molecular energies and HOMO/LUMO gaps^40,55,70,71^ and have been extended to the prediction of bond properties within molecules.^72^ In this work, our network was modeled after the *Schnet* deep learning architecture of Müller and coworkers,^67^ combined with edge updates.^73^ The model is implemented using *Tensorflow*, and all underlying code is openly accessible and documented.^74^ This was then trained to predict ^13^C and ^1^H chemical shifts as the target properties. A schematic of our network is shown in Fig. 1a. From a query 3D molecular structure, two input vectors are constructed with *rdkit*^75^ containing (i) element types and (ii) interatomic distances less than 5 Å. Discrete node feature vectors (of size 256) are then generated by categorizing each element type through an embedding layer, while continuous edge feature vectors are generated by an expansion of the interatomic distances as a series of 256 radial basis functions (RBFs).^73^ This is described by eqn (1), where the continuous vector 
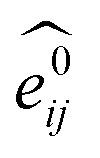
 represents the initial “edge” linking atoms *i* and *j* and is expressed in terms of the interatomic distance *d*_*ij*_ and constants *μ* and *δ*. These constants are chosen such that the range of the input features can be covered by the centers of the RBFs; in this work *δ* = 0.04 and *μ* = 0.1
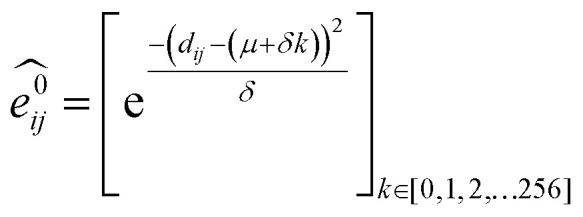


The feature vectors for atoms/nodes and bonds/edges then go through a loop consisting of edge updating, message passing, and node updating blocks (inset, Fig. 1a). In the message-passing block (brown color), each atom receives “messages” from other atoms within 5 Å, which reflect its local environment. We might reasonably expect to capture the shielding or deshielding influence upon chemical shift (whether these occur through-bond or through-space) of neighboring atoms, including those for which there is no direct bonding path. Using a larger cutoff distance led to a degradation in the model's validation loss (see ESI†). The final updated node feature serves as a 3-dimensional representation of the atomic environment for each atom, which is then passed through a fully connected NN^76^ to produce a chemical shift value. More details of the model architecture are provided in ESI Text 1.† Unlike models based only on atom-centered symmetry functions, our model allows local structural information to be exchanged between neighboring atoms. Chemical shift predictions for all atoms in the molecule are performed simultaneously, leading to an efficient numerical implementation.

## Learning DFT predicted chemical shifts

As an alternative to a large, manually curated collection of experimental chemical shifts, a computationally generated dataset offers several advantages. DFT computed chemical shifts are easily parsed and unequivocally assigned to the responsible atom in each compound. By sampling different structures, the dataset can be designed to ensure broad model coverage. Accordingly (Fig. 1b) we developed a dataset of 8000 DFT optimized structures with *ca.* 200 000 DFT computed chemical shifts (the *DFT8K* dataset). All datasets generated by this work are shared openly.^74^

We began by sampling a subset of structures from the NMRShiftDB, which contains 43 475 structures at the time of writing. The sampling procedure is as follows: we first extracted all neutral organic molecules with MW < 500. From the resulting set of around 20 000 structures, 8000 were selected by a farthest-neighbor algorithm^77^ to create a computationally manageable dataset while maximizing structural diversity.

Initial 3D geometries were then embedded from each molecule's SMILES representation using a distance geometry approach (ESI Text 2†),^78^ which was followed by conformational analysis with MMFF, culminating in the optimization of M06-2X/def2-TZVP geometries and empirically-scaled mPW1PW91/6-311+G(d,p) chemical shifts for each of these 8000 structures. This process was automated by a parallel Python workflow that takes structures from a 2D molecular database (NMR8K), performs conformational analysis, submits and monitors Gaussian jobs, and finally parses outputs (see ESI Text 2† for details on the automated workflow and DFT calculation methods). A new dataset, DFT8K, is populated by DFT optimized geometries and the corresponding computed chemical shifts (around 120 000 ^1^H and 100 000 ^13^C DFT chemical shifts in total, Fig. 1b). To obtain DFT-predicted isotropic chemical shifts we applied an empirical scaling formula to the raw shielding tensor values.^5,11^ The ^13^C chemical shift values were obtained from the relation *δ* = 181.40–0.97*σ* and ^1^H values from *δ* = 29.30–0.91*σ*.

**Fig. 2:**
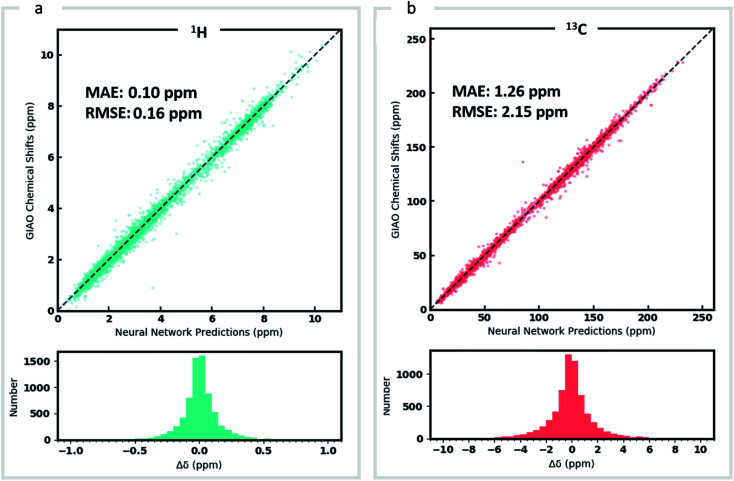
Prediction of DFT chemical shifts by the trained DFTNN model. Scatter plots and histograms compare DFT computations and GNN predicted chemical shifts for ^1^H (a) and ^13^C (b). The held-out test set contains 500 randomly sampled structures (testing/training rate: 1/12) from the DFT8K dataset.

DFT optimized geometries (inputs) and chemical shifts (prediction targets) from the DFT8K dataset were then used to train a GNN. 500 structures were used to evaluate the validation loss during model training, and another 500 structures were held-out as an external test set (Fig. 2). We refer to this ML model as DFTNN. Since ^13^C chemical shifts have a wider ppm distribution than ^1^H shifts we used separate models for each nucleus. DFTNN performs well in predicting the DFT shifts of held-out structures, giving a MAE and RMSE of 1.26 and 2.15 ppm, respectively, for ^13^C, and 0.10 and 0.16 ppm for ^1^H. These results compare favorably alongside other ML models for NMR chemical shift predictions. Kernel-based learning was reported to have an RMSE of 0.49 ppm for ^1^H and 4.3 ppm for ^13^C;^45^ a fully-connected neural network using HOSE descriptors^28^ has an RMSE of 2.7 ppm for ^13^C, and a 2D GNN based model has MAE of 0.22 ppm for ^1^H and 1.35 ppm for ^13^C.^79^ Direct comparisons are, however, complicated by the use of different training and test sets across different models.

## Transfer learning with experimental chemical shifts

Although DFTNN shows encouraging performance in predicting NMR chemical shifts, this GNN was trained solely against DFT calculated results that approximate experimental reality. Previous benchmarking studies suggest that DFT calculated chemical shifts have an RMSE of 0.1–0.2 ppm for ^1^H and 2.5–8.0 ppm for ^13^C, which vary according to functional and basis set used for the structure optimization and chemical shift calculation.^11^ To minimize prediction errors associated with the use of DFT reference data, we sought to further optimize performance by subjecting our GNN to additional refinement with TL, incorporating experimental data. Importantly, we also devised a strategy to check and clean these experimental data using the results of DFT calculations as described below.

**Fig. 3:**
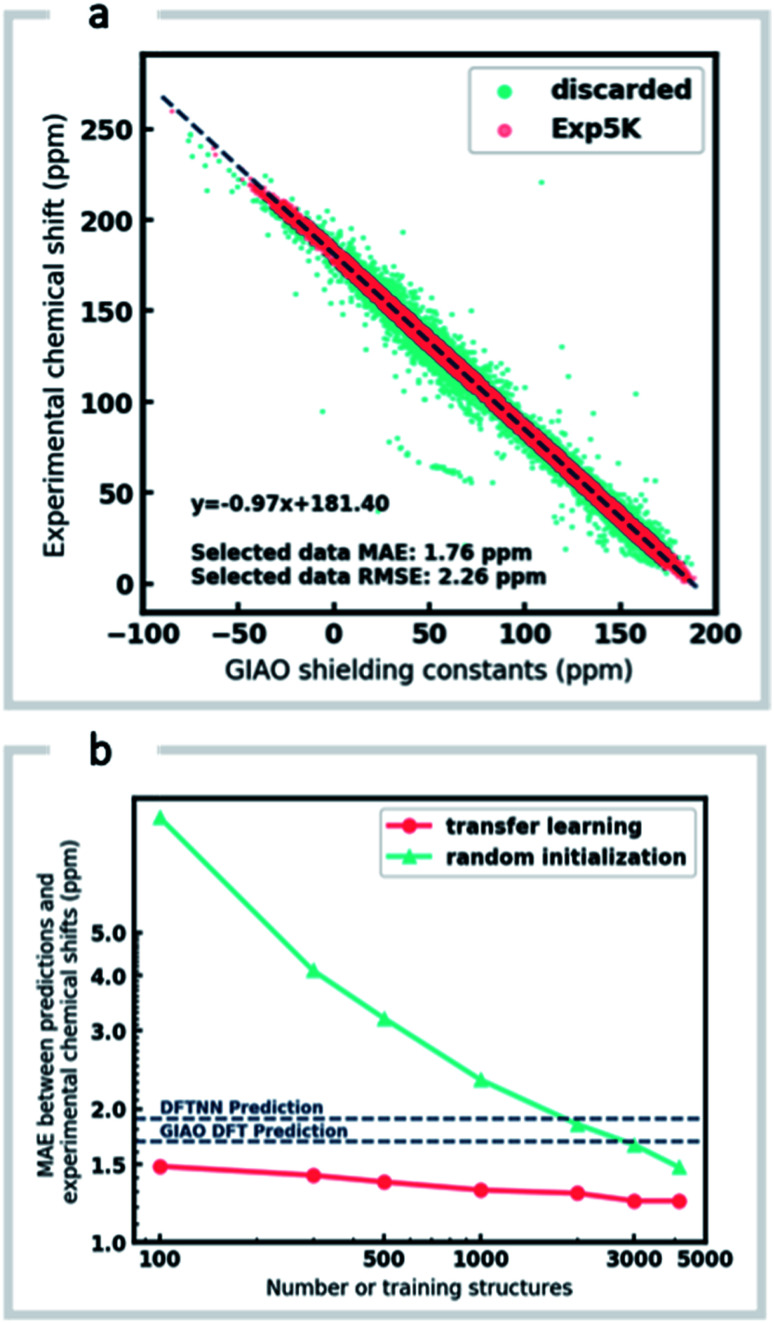
Learning experimental chemical shifts, (a) 53 334 DFT-computed and experimental ^13^C chemical shifts were compared to identify erroneous values. Outliers identified by IQR analysis (green) were removed while remaining data points (red) were retained and comprise the Exp5K dataset. (b) MAE of ExpNN-dft predictions against experiment as a function of training set size, with and without transfer-learning. The performance is also compared to DFTNN (green dash line) and DFT calculations (gray dash line).

Around 5500 molecules in the NMR8K dataset are annotated solely with experimental ^13^C data, while ^1^H and ^13^C chemical shifts are present for the remainder. ^1^H chemical shifts show greater sensitivity to the solvent used for experimental data collection, and while we had hoped solvent-induced variations in chemical shift could be captured during this next phase of model training, the identity of the solvent used was often lacking in our primary data. We were therefore forced to focus solely on the refinement of ^13^C predictions. We also had to disregard experimental data for structures with ambiguously defined stereochemistry. A more difficult task involves the removal of possible misassignments, for example where an experimental spectrum may be assigned to an incorrect structure or a chemical shift attributed to an incorrect atom.^29^ Since even a small fraction of anomalous training data can result in noticeable degradation of ML models,^45^ we adopted a cautious approach and rejected experimental data that was statistically at odds with our DFT calculations. A comparison of DFT and experimental ^13^C shifts (Fig. 3a) showed 911 values differing by >10 ppm (1.6% of all DFT calculated shifts) and 10% of values differing by >5 ppm. By removing outliers more than 1.5 interquartile ranges (IQRs) below the first quartile or above the third quartile, corresponding to 5% of the experimental data, the RMSE drops from 3.8 ppm to 2.26 ppm, which is close to the expected accuracy of our DFT methodology (2.4 ppm).^11^ Some of these discrepancies may reflect severe failings of DFT rather than errors in experimental assignments, however, the final performance of our model supports the use of this conservative strategy. Ultimately, this data-processing pipeline (ESI Fig. 6†) produced a “cleaned” dataset containing around 5000 structures and 50 000 experimental ^13^C chemical shifts, which we refer to as Exp5K.^74^

We then used transfer learning (TL)^59,80^ with the Exp5K dataset to retrain DFTNN. With TL, a pre-trained network model can be improved by learning from a new, higher accuracy dataset even when data is sparsely available.^60^ The optimizable parameters in our GNN model can be categorized into two groups: updating layers and the following readout layers (Fig. 1a). The updating layers learn how to encode atomic environments into an atomic fingerprint, while the readout layers interpret these fingerprints to generate chemical shift predictions. To preserve the information previously learned during model training against DFT results, as well as to prevent overfitting to the smaller Exp5K dataset, only the readout layers were optimized while the updating layers were frozen (Fig. 1b, with further details of implementation in ESI Fig. 1†). 500 molecules from Exp5K were held out as the test set. The resulting retrained model is named ExpNN-dft, since DFT optimized structures are still required as inputs. The ExpNN-dft predictions achieve a ^13^C MAE of 1.25 ppm and RMSE of 1.74 ppm for the held-out testing set. When compared with experimental chemical shifts, the accuracy of ExpNN-dft apparently surpasses that of DFTNN by more than 30% with a ^13^C MAE of 1.90 ppm.

We compared the above approach against training a model whose parameters are randomly initialized (*i.e.*, from scratch). Fig. 3b illustrates the efficiency of TL in the present work, and also highlights the fact that the performance of ExpNN-dft is superior to the DFTNN model and DFT computations, even though the experimental training set is relatively sparse. The success of this approach arises from the strong correlation between DFT chemical shifts and experimental shifts, the molecular structures shared by DFT8K and Exp5K, and the strategy of freezing 94% of GNN hyperparameters during TL.

## Transfer learning to use inexpensive molecular geometries

Our GNN models give rapid NMR chemical shift predictions, which through the inclusion of experimental training data, outperform DFT accuracy. However, the requirement of DFT optimized structures as inputs significantly limits a model's practicality and applicability. Therefore, we opted to retrain the ExpNN-dft model using 3D structures obtained from inexpensive molecular mechanics (MM) calculations (MMFF94)^81^ as input, retaining experimental chemical shifts from Exp5K as targets. Transfer learning was again employed for this retraining. This time, however, to reflect the fact that the training data contains modified molecular geometries, the six hidden layers in the edge updating block were optimized (Fig. 1b), while all other parameters were held fixed. This second round of transfer learning led to a ^13^C MAE of 1.43 ppm against experiment. This final GNN model, named ExpNN-ff, retains the high accuracy of the previous models while processing MM input structures, facilitating real-time ^13^C chemical shift prediction.

**Fig. 4:**
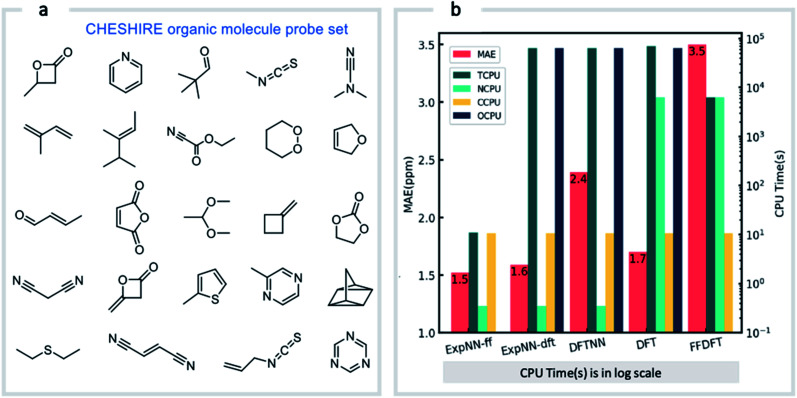
GNN performance on the CHESHIRE set of organic molecules. Performance and computational cost for three GNN models (ExpNN-ff, ExpNN-dft, and DFTNN) and DFT methods (DFT and FFDFT) for the CHESHIRE testing set.^45^ DFT indicates optimizations and chemical shift prediction at this level, while FFDFT indicates DFT shift predictions on MMFF geometries. CPU times are shown in logarithmic scales. TCPU: total CPU time of computing chemical shifts from smile strings for CHESIRE testing set; NCPU: CPU time for NMR chemical shift computations; CCPU: CPU time for conformer searching through MMFF94; OCPU: CPU time for structure optimizations.

The three trained GNN models (DFTNN, ExpNN-dft, and ExpNN-ff) were evaluated using an external dataset of chemical shifts, CHESHIRE, which is widely used to benchmark DFT methods (Fig. 4a). ExpNN-ff, which avoids expensive DFT structure optimizations, took 10 seconds of CPU time to predict all ^13^C chemical shifts for 24 molecules in the CHESHIRE test set compared to 19 hours for those methods requiring DFT structure optimization. Note that the GNN model in the ExpNN-ff workflow only cost 3% of the total CPU time (0.35 s), while the highest cost is still on conformer searching. Even though using MMFF structures as inputs, the performance of ExpNN-ff does not degrade compared to ExpNN-dft (Fig. 4b). In contrast, performing DFT chemical shift predictions on MMFF geometries (FFDFT),^18,82^ leads to a noticeable degradation in performance for this testing set. Out of 25 electronic structure methods mPW1PW91/6-311+G(2d,p)//M062X/6-311+G(2d,p) calculations provide the lowest MAE for this dataset (ESI Table 2†), however, all are outperformed by our two GNN models augmented by transfer learning against experimental data. Of these, ExpNN-ff is around four orders of magnitude faster. Encouraged by this comparison against DFT methods that have been applied successfully to revise organic structures,^3–5^ we next set out to apply whether the ExpNN-ff model can be accomplish more challenging applications of structure elucidation in seconds.

## Application to structure elucidation and reassignment

**Fig. 5:**
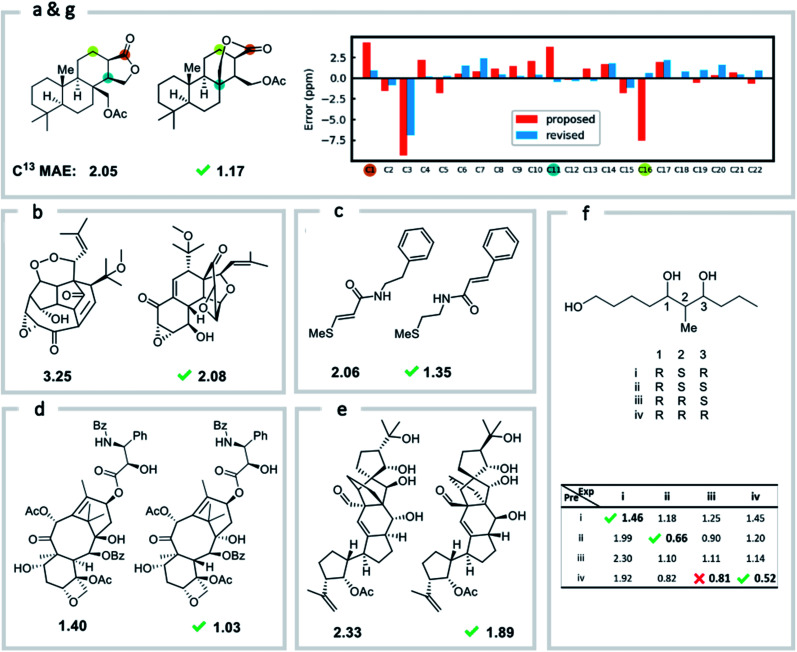
Structure elucidation using ExpNN-ff. (a)–(e) Historical cases of natural product structural misassignment. MAE values are compared for the originally proposed, but incorrect, structure and the revised, correct structure against experimental ^13^C spectra. In each case a better match is obtained for the correct structural assignment in seconds. (f) MAE values obtained by comparing all four diastereomeric structures of a highly-flexible 1,3-diol against four sets of experimental data. In three of four cases the lowest MAE value matches the correct spectrum. (g) The error between predicted and experimental chemical shifts for each atom in proposed and revised structures for example (a).

We first confirmed the ability of ExpNN-ff to describe stereochemical and conformational effects upon chemical shift. We were pleased to see that for the three cases outlined in Scheme 1, our approach was able to (a) successfully discriminate between the diastereomers of 1,3-hydroxymethylcyclohexane, (b) predict different chemical shift values for the diastereotopic methyl groups of l-valine, and (c) show differences between the two conformers of methylcyclohexane (quantitative comparisons are shown in ESI Text 6†). Importantly, in each case the use of a conventional HOSE-based or 2D graph approach would be unable to provide any such distinction. We then turned to significantly more challenging tasks of structure elucidation, several of which would be extremely taxing for conventional DFT-based approaches due to their complexity in terms of size and conformational flexibility (Fig. 5a–f). Constitutional isomers are compared in the first three examples, while the final two involve pairs of diastereomers. For cases (a–e), we compare the predicted chemical shifts for two candidate structures against the experimental 13C spectrum. All analyses are automated from SMILES queries, with sorted lists of predicted and experimental shifts being compared. ExpNN-ff gives a lower MAE for the correct assignment across all five examples. A detailed breakdown for (a) is shown in Fig. 5f, in which the most egregious errors of the originally proposed, incorrect assignment (*e.g.*, at C1, C11, and C16) are highlighted. Predicted chemical shifts for these atoms in the revised, correct structure are much closer to the experimental data. We further tested ExpNN-ff to match the four diastereoisomers of a conformationally flexible 1,3-diol with four experimental NMR spectra (Fig. 5f). Since ExpNN-ff generates conformer-specific predictions (ESI Fig. 8†), these were Boltzmann weighted (using MMFF relative energies) from around 200 conformers to yield final predictions. The lowest MAE was obtained for the correct diastereomer in three out of four cases. However, ExpNN-ff could still be used to correctly assign all four diastereoisomers by considering the cumulative MAE values across all structures.

**Fig. 6:**
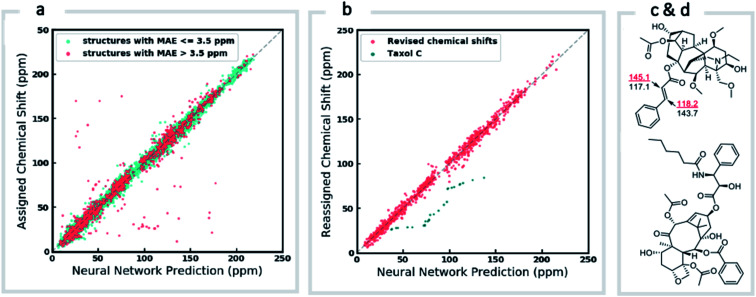
Screening and revising misassignment in NMRShiftDB. (a) Correlation between predicted and experimental ^13^C chemical shifts for large molecules (MW > 500). Outliers (red), here defined as structures with an MAE > 3.5 ppm, are investigated for possible misassignments (b) experimental chemical shifts for reordered assignments of outlying structures. The remaining outliers (green) helped us to identify an incorrect structure for Taxol C in the database. (c) Incorrectly assigned enoate carbons were corrected for leueantine A. (d) The correct structure of Taxol C.

We next investigated the performance of the ExpNN-ff model for organic structures larger than those used for network training (MW > 500). We compared our predicted ^13^C chemical shifts against experimental values for 650 large molecules (MW > 500) taken from NMRShiftDB (Fig. 6a). Each prediction requires at least one MMFF conformation of a given molecule and where multiple conformers were present a Boltzmann-weighted average was used. As an illustrative example, we used ExpNN-ff's predictions to detect obvious database errors/misassignments in an automated, high-throughput fashion. Predicted chemical shifts were first compared against the structural assignments from NMRShiftDB. For structures with MAE values >3.5 ppm the experimental shift values were reordered to find the optimal assignment (*i.e.*, lowest MAE, Fig. 6b). One such example automatically identified is shown in Fig. 6c, where enoate α- and β-carbon shifts were found to be swapped in the experimental assignment. After this workflow was complete, remaining egregious outliers were then inspected manually. The structure of Taxol C (ID: 20244313) was found to be incorrectly recorded in the database, with a cyclohexyl rather than phenyl ring. This approach highlights the application of ExpNN-ff as high-throughput method to detect assignment errors, however, the incorporation of sophisticated metrics such as Goodman's DP4 (ref. 18) would be necessary for a more rigorous evaluation of possible structural assignments, and is the subject of further work.

## Application as atomic descriptors in selectivity prediction

**Fig. 7:**
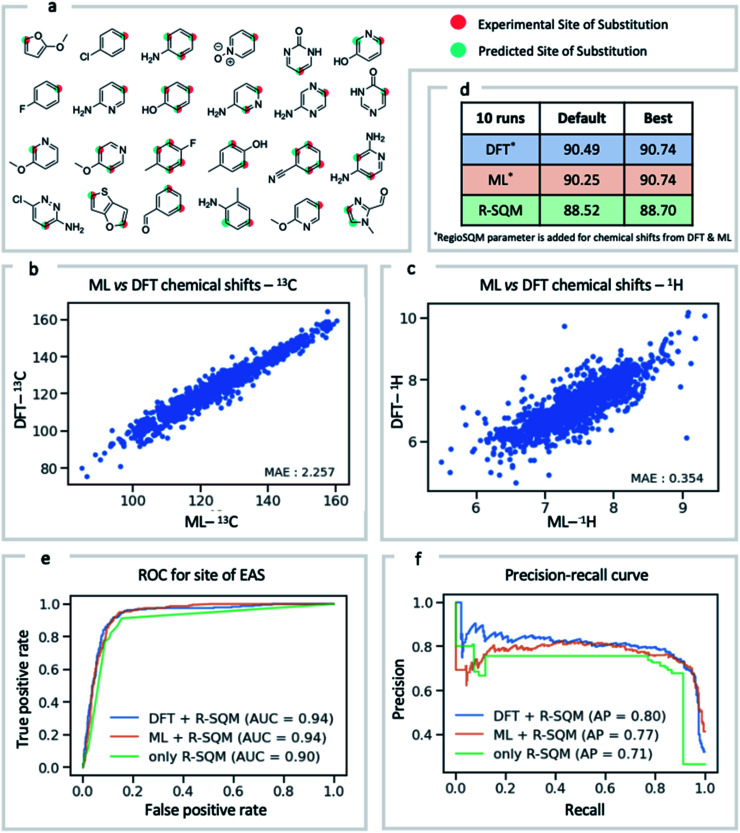
Regioselectivity prediction of electrophilic aromatic substitutions. (a) Representative molecules present in the EAS dataset. The highlighted atoms depict the experimental (red) and the predicted (green) site of substitution. (b) DFT computed ^13^C chemical shifts *vs.* GNN-predictions. (c) DFT computed ^1^H chemical shifts *vs.* GNN-predictions. (d) Random forest classifier accuracies in identifying reactive/unreactive ring positions. (e) ROC curves comparing the true positive *vs.* false positive rate. (f) Precision–recall curves for the different random forest classifiers.

NMR chemical shift is influenced by the electron density around a nucleus of interest. It is therefore an attractive choice of physically-motivated and interpretable atomic descriptor for use in predictive machine learning models.^83,84^ By foregoing expensive quantum chemical computations, chemical shifts accurately predicted by ExpNN-ff provide easier and faster access to descriptors for use in regression tasks such as reactivity and selectivity prediction. We have investigated this approach in predicting the regioselectivity of electrophilic aromatic substitution (EAS) reactions. Previously, the combination of DFT-computed atomic Fukui coefficients, atomic partial charges, bond orders, and partitioned solvent-accessible surface areas with semi-empirical regioSQM^85^ predictions was used to develop a random forest (RF) model with 93% accuracy in predicting the site of substitution using 80/20 train/test splits for 376 molecules.^86^ Below (Fig. 7) we demonstrate comparable accuracy with fewer atomic descriptors, using just (i) the ^13^C chemical shift, (ii) the attached proton ^1^H chemical shift, and (iii) the regioSQM prediction. We also find that using GNN predicted shifts gives similar performance in place of more expensive DFT (mPW1PW91/6-311+G(d,p)//M062X/def2TZVP) values. The prediction accuracy averaged across 10 runs for different RF models is shown in Fig. 7d. After optimization of model hyperparameters, accuracy increases with the inclusion of chemical shift descriptors to 90.7% from 88.5% using regioSQM alone. ROC and precision–recall plots (Fig. 7e and f) illustrate that the inclusion of chemical shift descriptors increase the performance of an RF classification (*i.e.*, correctly labelling reactive and unreactive positions) from 0.90 to 0.94 and that the average precision is also higher with chemical shift descriptors. These GNN-derived atomic descriptors impose low computational cost such that we anticipate future utility in related prediction tasks of organic reactivity and selectivity, for example in combination with other machine-learned representations.^87^

## Conclusion

Predicting NMR chemical shifts in real-time that can distinguish stereoisomers and configurations/conformations poses both conceptual and technical challenges. The GNN model we have presented in this work overcomes this hurdle by learning suitable atomic environments from 3D structures and predicting chemical shifts based on these learned environments. MAEs between GNN predicted chemical shifts and DFT are 0.16 ppm for ^1^H and 1.26 ppm for ^13^C, which compare favorably with other approaches. This approach requires large quantities of labelled chemical shift data, which was provided by a large-scale quantum chemical dataset. To mitigate errors associated with using DFT training data, we also curated a smaller dataset of experimental chemical shifts that was used for retraining the NN model through transfer learning. Additionally, the model was retrained to process inexpensive molecular mechanics 3D geometries so that high-quality structures are not a prerequisite. These steps resulted in a predictive model of comparable accuracy to DFT when compared against experimental chemical shifts of small organic molecules, with a 7000-fold performance increase. This efficiency enabled us to (i) perform GNN ^13^C predictions for flexible structures impractical to study with DFT with sufficient accuracy to discriminate between correct and incorrect assignments, (ii) carry out high-throughput screening and error detection of a large database of NMR assignments and (iii) rapidly obtain chemical shifts to be used as atomic descriptors in a machine learning model for regioselectivity. The resulting deep learning model can be used as a command line tool or as a web-based product-level calculator that allows real-time chemical shift predictions from a molecule sketch or SMILES input (http://nova.chem.colostate.edu/cascade/predict/).

Just as every model has limitations, the framework we present in this work still leaves room for improvement. We mention that the accuracy of the model depends on the quality of 3D structures generated by MMFF to some extent. We have found several examples where the poor MMFF structure leads to a discrepancy in prediction, for instance, ketenimines. Thus, the model is likely to improve further with more robust empirical or semi-empirical structures, along with associated relative energies that are used to carry out Boltzmann averaging, such as those from xTB.^88^ Other potential improvements will include extending the model to biomolecules, coupling constant prediction, and the adoption of probability metrics such as DP4 for structure elucidation.

## Methods

### Computational details

NMR isotropic chemical shifts in the present work are predicted using a GNN derived from *Schnet*.^40,67,73^ The network receives 3D molecular structures *via* a vector of atom types and a vector of interatomic distances. The network is directly trained against chemical shifts for individual atoms. As discussed above, these chemical shifts are sourced from empirically-scaled DFT computations and this training data is augmented by experimental values during later stages of model training. Atom indices are also processed by the neural network, which is used to pool out corresponding node features in the readout layer. Detailed architectures, hyper-parameters, and training processes are given in the ESI Section 1.†

Three subsets of organic structures from the NMRShiftDB are used in this work, referred to as NMR8K, DFT8K, and Exp5K. The NMR8K dataset contains 8000 neutral molecules with molecular weights up to 500, comprising elements: C, H, O, N, F, Cl, P, S. 3016 of these structures have associated ^1^H NMR experimental spectra; 6000 have associated ^13^C spectra. These structures were processed with a computational workflow to generate the DFT8K dataset used for our GNN training. Our workflow involved embedding and molecular mechanics (MM) conformational analysis with the MMFF94 force field implemented in *rdkit*.^81^ The most stable MM conformers were then optimized at the M06-2X/def2-TZVP^89^ level of theory, for which isotropic shielding constants were then calculated with gauge-independent atomic orbital (GIAO)^90^ method at the mPW1PW91/6-311+G(d,p)^91^ level of theory. This combination of MM and DFT methods has been used successfully for structure assignments with NMR chemical shift predictions.^92^ This workflow produced 7455 DFT optimized structures with 117 997 ^1^H and 99 105 ^13^C calculated chemical shift values, which make up the DFT8K dataset. The NMR8K and DFT8K datasets were then compared to prepare a clean experimental dataset from which apparent outliers are absent. This produced 5631 structures labeled with 59 413 experimental ^13^C chemical shifts, which make up the Exp5K dataset. Further details of dataset construction are contained in the ESI Section 2.†

Three separate GNNs were trained, referred to as DFTNN, ExpNN-dft, and ExpNN-ff. Architectures and hyper-parameters for these networks are the same, but they are trained against different targets or using different input structures. The DFTNN is trained against DFT calculated chemical shifts using the optimized geometries from the DFT8K dataset with randomly initiated parameters. This model is then retrained against experimental chemical shifts from the Exp5K dataset while retaining the DFT geometries, with partially fixed parameters to generate the ExpNN-dft model. Finally, the model is again retrained using experimental chemical shifts from Exp5K while geometries are replaced by MMFF structures, with partially fixed parameters to produce the ExpNN-ff model. Further details on transfer-learning and frozen parameters are given in the ESI Section 3.†

### Practical usage considerations

All code is openly accessible from GitHub under an MIT license at https://github.com/patonlab/CASCADE. This includes the automated workflow to process a SMILES query, perform conformational analysis and 3D structure optimization, and generate NMR chemical shift predictions, as well as the three ML models (DFTNN, ExpNN-dft, and ExpNN-ff) presented here. Training and testing data for each deep learning model are also publicly available from the same GitHub repository. For ease of use, a real-time web-app has been developed, http://nova.chem.colostate.edu/cascade/predict/which performs ^1^H and ^13^C predictions for SMILES queries or *via* a graphical molecular editor. Boltzmann averaged and individual conformer-specific chemical shifts are rendered with *JSmol*.

## Data availability

The Python workflow, final trained models, raw and processed datasets, and analyses are freely available under an MIT license at https://github.com/patonlab/CASCADE.

## Author contributions

Y. G. designed the machine learning architecture, implemented the high-throughput DFT calculations, and trained the neural networks under the guidance of P. C. St. J. and R. S. P. S. V. S. S. and L. C. G. carried out the regioselectivity predictions. R. S. P. conceived the study. All authors participated in editing the final paper.

## Conflicts of interest

There are no conflicts to declare.

## Supplementary Material

SC-012-D1SC03343C-s001
