# Supplementary material for: Real-time prediction of 1H and 13C chemical shifts with DFT accuracy using a 3D graph neural network
Source: Chem Sci. 2021 Aug 9;12(36):12012–26. doi: 10.1039/d1sc03343c (PMC8457395; doi:10.1039/d1sc03343c)
Supplement: SC-012-D1SC03343C-s001 [file SC-012-D1SC03343C-s001.pdf]

Electronic Supplementary Material (ESI) for Chemical Science.
This journal is © The Royal Society of Chemistry 2021

Supplementary Information

Real-time Prediction of ^{1}H and ^{13}C Chemical Shifts with DFT accuracy using a 3D Graph Neural Network

Yanfei Guan[†*], Shree Sowndarya S. V[†], Liliana C. Gallegos[†], Peter St. John[‡], Robert S. Paton[†*]

[†]Department of Chemistry, Colorado State University, Fort Collins, CO, 80523, USA
[‡]Biosciences Center, National Renewable Energy Laboratory, Golden, CO 80401, USA
Corresponding e-mail: yanfei.guan@pfizer.com; robert.paton@colostate.edu

Table of Contents

SI Text 1. Graph convolutional network..1

SI Figure 1. Architecture of the graph convolutional network..................3

SI Figure 2. Optimizing hyperparameters of the network.........................3

SI Figure 3. Message flow in the graph network 4

SI Text2. Constructing Databases...4

SI Figure 4. Workflow for DFT computations..5

SI Figure 5. Distribution of calculated shielding constants.......................5

SI Figure 6. Cleaning NMR8K database...6

SI Figure 7. Chemical shift distribution of the Exp5K...............................7

SI Text 3. Transfer learning to experimental chemical shifts.....................7

SI Figure 8. Performance of ExpNN-*dft*...7

SI Text 4. Transfer learning to use inexpensive molecular geometries......8

SI Figure 9. Performance of ExpNN-*ff*...8

SI Text 5. Testing on the CHESHIRE testing set......................................8

SI Figure 10. CHESHIRE testing molecules..9

SI Table 1. Chemical shifts calculated for CHESHIRE molecules..........10

SI Table 2. ExpNN-*ff* *vs* QM methods..13

SI Text 6. Quantitative comparisons for three examples in scheme 1......14

SI Text 7. Structure assignments via ExpNN-*ff*......................................16

SI Text 8. Conformer based predictions for flexible molecules..............26

SI Figure11. Conformer based predictions for flexible molecules..........26

SI Text9. Chemical shifts revision for large molecules............................27

Supplementary Information

Supplementary Information Text 1 | Graph Convolutional Network

1.1. Embedding 3D structures. Isotropic NMR chemical shifts for ^1H and ^{13}C are predicted using a graph convolutional network (GCN). The detailed architecture of this network is given in Supplementary Figure 1. Four types of tensor are taken as input:

(i) nodes, which encode atom types

(ii) edges which encode interatomic distances

(iii) targets, the 1H or 13C values to be predicted, which are put in as atom indices

(iv) connectivity information, which encodes ends of each edge, esp. starting and ending atoms for the directional edge.

Node features, h^0 of size 256 are initiated through categorizing atom types. Edge features e^0 with size 256 are initiated by expanding interatomic distances in a radial basis sets[1]:

$$\widehat{e_{ij}^{0}} = [\exp(-\frac{(d_{ij} - (\mu + \delta k))^2}{\delta})]_{k\in[0,\,1,\,2,\ldots256]}$$

Where e_{ij}^0 is the initial feature vector for the edge connecting atoms *i* and *j*. d_{ij} is the interatomic distance between atoms *i* and *j*. μ and δ are chosen such that the range of the input features are encoded by the centers of these functions. Herein we choose δ as 0.04 and μ as 0.

Note that not all interatomic distances are embedded as edge features. A distance cutoff d_c excludes remote atom pairs. Optimization of this distance cutoff value is described in SI **1.6**

1.2. Edge updating. The embedded edge feature e_{ij}^0 is updated in the edge updating blocks by concatenating edge features with node features of the starting and ending atoms (atom ***i*** and atom ***j***)[2]:

$$e_{ij}^{t+1} = \sigma\left(W_4^t \sigma W_3^t W_2^t \sigma\left(W_1^t\left(h_i; h_j; e_{ij}^t\right)\right)\right) + e_{ij}^t$$

Where $\{W_1^t, W_2^t, W_3^t, W_4^t\}$ denote trainable weight matrixes, σ is soft-plus activation function, and (;) refers to the concatenation operation.

1.3. Message passing. Updated edge feature e^{t+1} are then used to generate a message passed to each individual atom in the message passing block. For instance, atom *j* receives messages from surrounding atoms within a distance cutoff by:

$$m_j^{t+1} = \sum_{i\in N} W_5^t h_i^t \circ e_{ij}^{t+1}$$

Where $\{W_5^t\}$ is trainable weight matrixes, $\circ$ is the element-wise product, and h_i^t is the current node feature vector for atom *i.* The updated edge e_{ij}^{t+1} can be interpreted as 256 continuous filters based on interatomic distances as well as sending and receiving atoms, which corresponds to the discrete filters employed in convolutional neural networks for image processing. The element-wise product can be interpreted as a convolution between atom ***i*** and corresponding filters to yield a message passed from atom *i* down to atom *j*. All messages flowing to atom ***j*** will then be pooled into a single message m_j^{t+1}.

1.4. Node updating. The message received by atom j is added to the current node feature vector, updating the node:

$$h_j^{t+1} = h_j^t + W_7^t \sigma\left(W_6^t m_j^{t+1}\right)$$

1.5. Readout. Node features and edge features are updated three times through the above process. The final node features involving spatial and chemical environment information will pass through a series of dense layers to give the final chemical shift predictions.

1.6. Hyperparameter selection. The graph network model in the present work contains several adjustable hyper-parameters, as discussed in reference 3. These hyperparameters include the distance cutoff d_c, the size of edge and node features, and the number of updating loops. A small feature vector size cannot capture all necessary information, while too large feature vectors can lead to redundant descriptions, resulting in overfitting as well as an unstable model. We optimized the feature vector size by gradually increasing it until reaching a best performance regarding to the loss function for the dev set (validating set), which was obtained for vectors of length 256.

The distance cutoff d_c plays an important role in the network accuracy and speed. We compare the performance of the *DFTNN* network model on the testing set as a function of distance cutoff (SI Fig. 2a). The optimal distance cutoff d_c was selected as 5 Å.

The updating layers, colored by gray shadow in SI Fig. 1, are repeated several times to update the node and edge features. As shown in SI Fig. 2b, the mean absolute error (MAE) on validating set reaches minimum point for three updating loops.

The distance cutoff and number of updating loops influence how the network captures the spatial and chemical environments in a given molecule. For each individual atom, the distance cutoff determines the radius for a local atomic environment to be involved, while the updating layer loops allow indirect communication with a distant atom through intermediate atoms, allowing the network to capture global impacts. For example, such spatial and chemical environments are depicted in SI Fig. 3. In the limit of a single updating loop, information passed by the network is highly local, resulting in poor performance.

1.7. Implementation. The graph convolutional network is implemented using Keras[4] and Tensorflow[5]. Scikit-learn[6] is used to scale prediction targets. RDKit[7] is used to extract interatomic distances and to encode the atoms as integer classes. The model was trained on a single GPU (Tesla K80) with a batch size of 32. Models were optimized using the Adam first-order gradient-based optimizer[8] and MAE loss function. The learning rate of 5×10^{-4} and explicit learning rate decay of 4% every 70 epochs was used. Models were trained for 1200 epochs. Early stopping was employed by evaluating the validation loss every 10 epochs and using the model that yields that lowest validation loss.

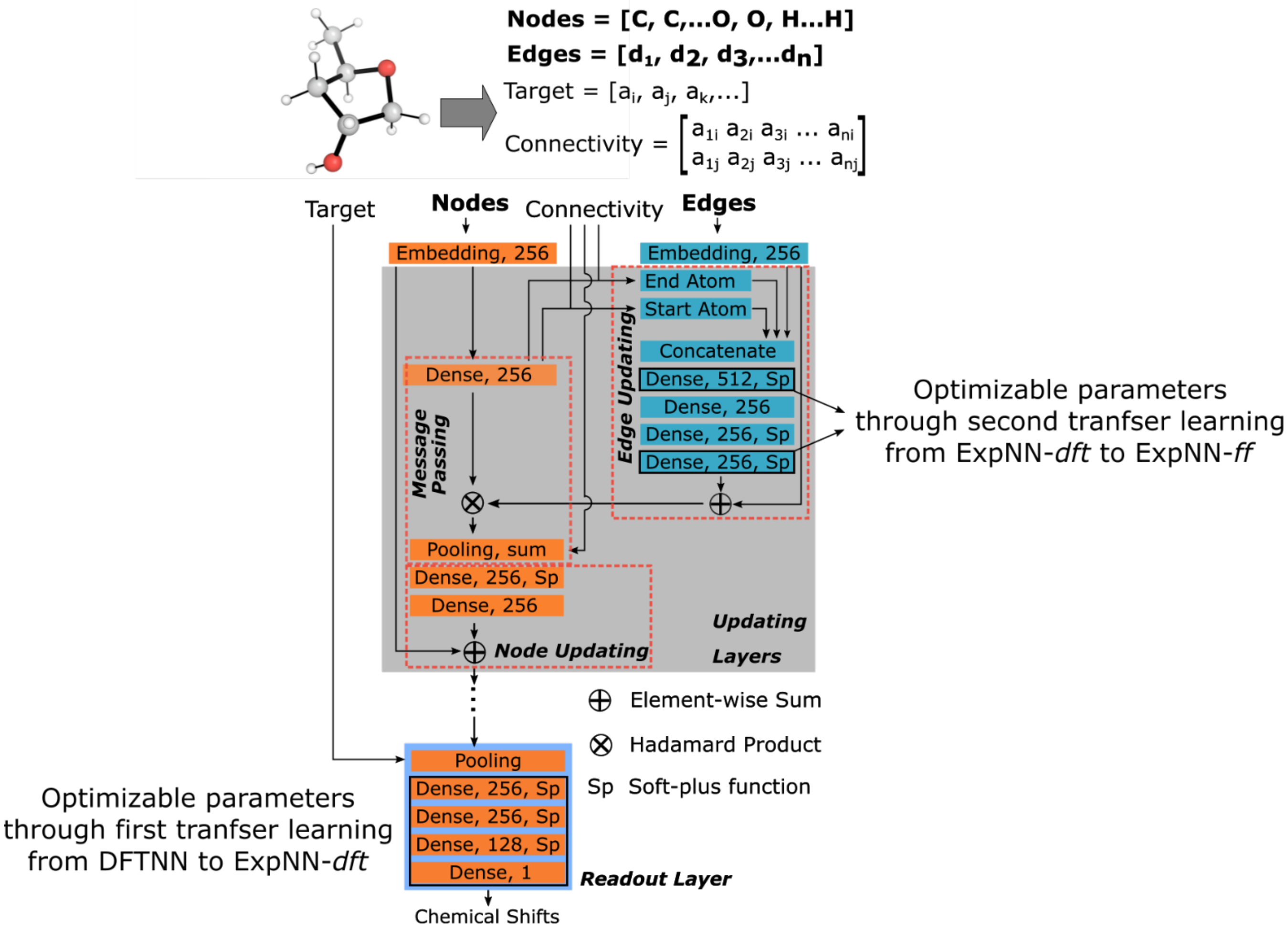

Supplementary Information Figure1. Detailed architecture of the graph convolutional network. Layers with a black border contain parameters that were further optimized by transfer learning, during which all other parameters are fixed.

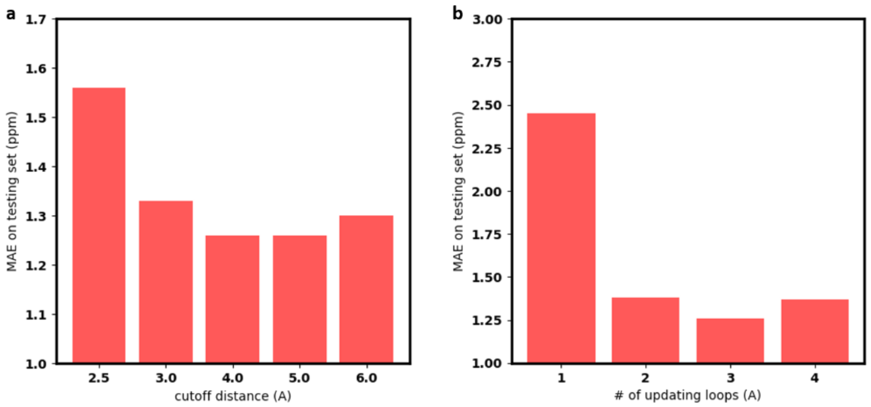

Supplementary Information Figure2. Optimizing hyperparameters for the graph network. **(a)** Performance on testing set as a function of distance cutoff. Three updating loops were used. **(b)** Performance on testing set as a function of the number of updating loops. A cutoff distance of 5 Å was used.

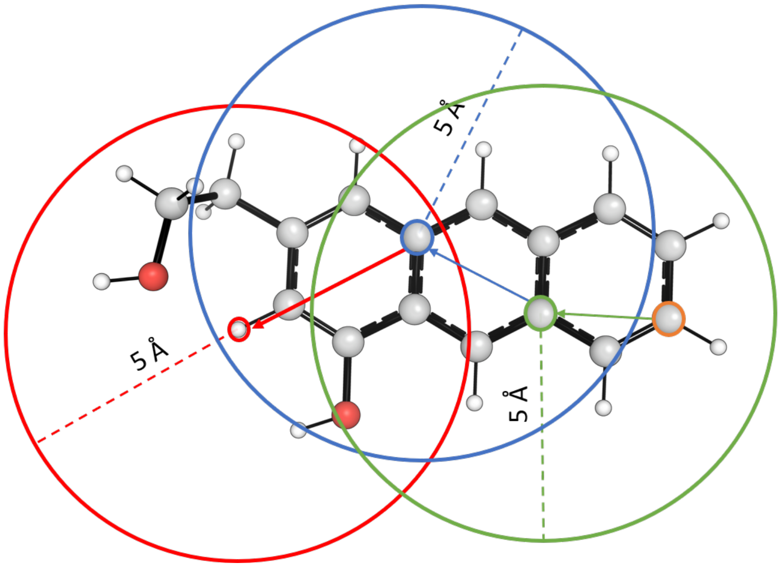

Supplementary Information Figure 3. Message flow in the GCN. Each circle denotes a spherical volume in which atoms can directly pass a message to the central atom, highlighted by a smaller circle with the same color. Information from the carbon atom colored in orange flows to the H atom (in red) through three updating layers – one such path is shown via the intervening green and blue carbon atoms. According to this approach, the influence of both the immediate local atomic environment (red circle) and more remote environment (blue big circle and green big circle) are captured in the description of each atom.

Supplementary Information Text 2 | Database Construction

2.1. 2D structures. All 2D structures were obtained from the NMRShiftDB database.[9] A total of 4,3425 structures was downloaded from the NMRShiftDB. We applied the following rules to select structures:

1. Molecular weight of the structure should be < 500.
2. The structure must contain at least one carbon atom.
3. The structure can only contain C, H, O, N, P, S, F, and Cl atoms
4. The molecule must be neutral.

Using these rules, we extracted 1,8414 molecules. A farthest neighbor algorithm[10] was then applied to sample a subset:

2.2. Farthest neighbor sampling. In order to cover as uniformly as possible, the structural space of the database and thus to provide a general dataset, a farthest neighbor sampling algorithm is implemented. The first structure is selected randomly, and the others in the sequence are picked to minimize:

$$S = \sum_{i,j \in sample}^{i \neq j} s_{ij}$$

Where d_{ij} is the Tanimoto similarity between molecules computed using Morgan fingerprints.[11] The sampled 8,000 molecules along with associated experimental chemical shifts comprise the NMR8K dataset.

2.3. DFT Calculations for 3D structures and shielding constants. We implemented an automated workflow for the high-throughput calculation of 3D structures and GIAO DFT shielding constants from 2D structures obtained in **2.2**. The workflow is schematically illustrated in SI Fig. 3. The workflow evenly divides the NMR8K into multiple subsets. For each subset, the workflow performs 3D structure embedding, conformer searching, DFT optimization, structure checking, and GIAO DFT calculations for each molecule. Conformers for each molecule were generated through the distance geometry method with 1000 initial random conformers. The MMFF94s force field[12] was used to minimize the energy for each conformer. Both the distance geometry method and MMFF94s force field were used as the built-in function of RDKit. The lowest lying conformer from force field minimization was then selected for further optimization with density functional theory (DFT). Gaussian 16[13] was used for all electronic structure calculations. 3D structures for these molecules are optimized at the M06-2X/def2-TZVP level of theory.[14] GIAO (gauge-including atomic orbitals) shielding constants were calculated at the mPW1PW91/6-311+G(d,p) level of theory, which has been shown to provide high accuracy for NMR calculations.[15] The presence of imaginary frequencies was detected, and re-optimizations were launched automatically until reaching an energy minimum (zero imaginary frequencies) or the maximum number of optimization steps was exceeded. The workflow monitors these processes and collates results automatically. Such calculations were performed in parallel for all subsets on multiple CPU nodes, while within each subset calculations are carried out sequentially for each molecule. Using this workflow, we obtain 7,455 optimized structures along with 117,997 ^{1}H and 9,9105 ^{13}C calculated chemical shifts, which compose the DFT8K database.

The distribution for computed shielding constants for ^{1}H and ^{13}C are shown in SI Fig. 4

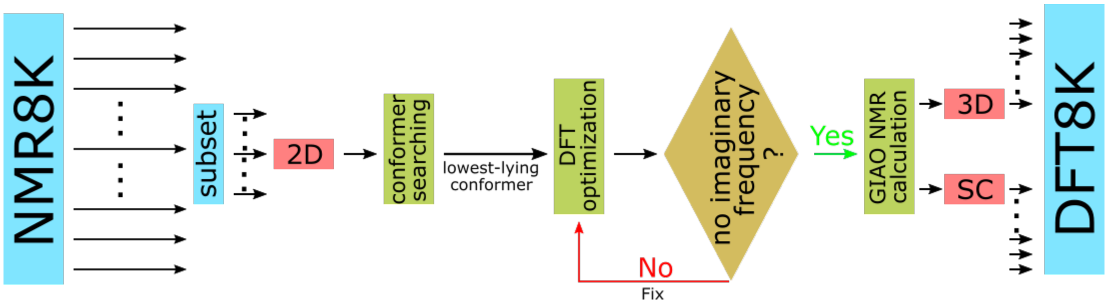

Supplementary Information Figure 4. Schematical illustration for workflow generating 3D structures and calculated shielding constants (SC).

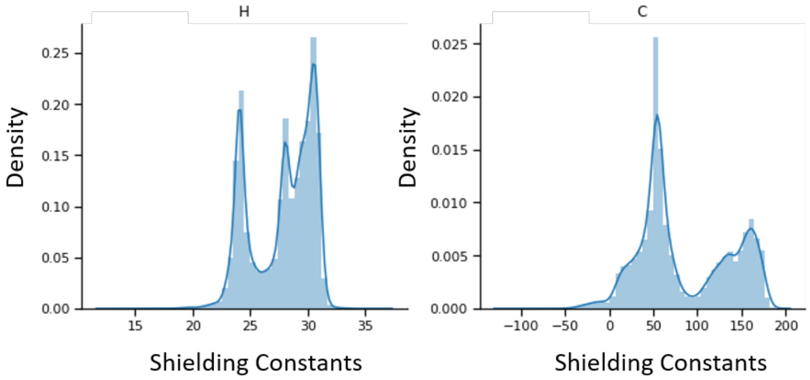

Supplementary Information Figure 5. Distributions of mPW1PW91/6-311+G(d,p)//M06-2X/def2-TZVP calculated $^{1}H/^{13}C$ shielding constants for DFT 8K. There are 117,997 ^{1}H and 9,9105 ^{13}C calculated chemical shifts.

2.4. Cleaning the experimental chemical shifts with GIAO DFT calculations. Our transfer learning strategy is reliant upon access to several thousand experimental chemical shifts. These were taken from NMRShiftDB. The assignment of experimental NMR spectra to chemical structures, and of the chemical shifts to individual atoms, is generally performed manually. Erroneous structures and assignments are encountered in the scientific literature, and we cautiously assumed that this may also be possible within our chosen experimental data source. For this reason, we used the presence of statistical outliers in these data (when compared against the chemical shift predictions of DFT calculations) to remove potentially erroneous assignments. For example, extreme disagreements between experiment and DFT (e.g., $\Delta\delta$ values > 20 ppm for ^{13}C) are rare, and generally indicative of incorrect assignments. However, there is a trade-off to be made here, since some of the larger errors may also be due to failures of DFT, and the inclusion of such points would be advantageous for the transfer learning stage of model training. We used a statistical definition of outliers in terms of the interquartile range (IQR), discarding data with DFT vs. experimental error below (Q_1 – 1.5*IQR) or above (Q_3 + 1.5*IQR). This corresponds to -6.1 and 5.9 ppm, respectively. As a result, about 8% of data was discard, leaving 53,331 ^{13}C chemical shifts. Discarded datapoints are accessible from GitHub (https://github.com/patonlab/CASCADE).

Experimental chemical shifts collected in **2.1** and **2.2** were checked and cleaned by GIAO DFT calculated shielding constants obtained in **2.3**. In the ideal case, chemical shifts δ can be calculated from GIAO DFT shielding constants σ by:

$$\delta = \sigma_{ref} - \sigma$$

where σ_{ref} is the calculated shielding constant of a reference sample, for example the ^{1}H or ^{13}C nuclei in tetramethylsilane (TMS). However, an alternative and generally more accurate approach is to obtain chemical shifts δ by applying empirical linear-scaling relationships[16] obtained from correlation between DFT and experiment. Herein, we use such a relationship to check for possible incorrect assignments of experimental chemical shift based on the presence of statistically significant outliers.

The process of checking and discarding incorrect assignments is shown in SI Figure 5. The remaining data are constructed into a database named Exp5K, which containing 5148 structures and 53334 ^{13}C chemical shifts. The distribution of experimental chemical shifts in this database is shown in SI Figure 6.

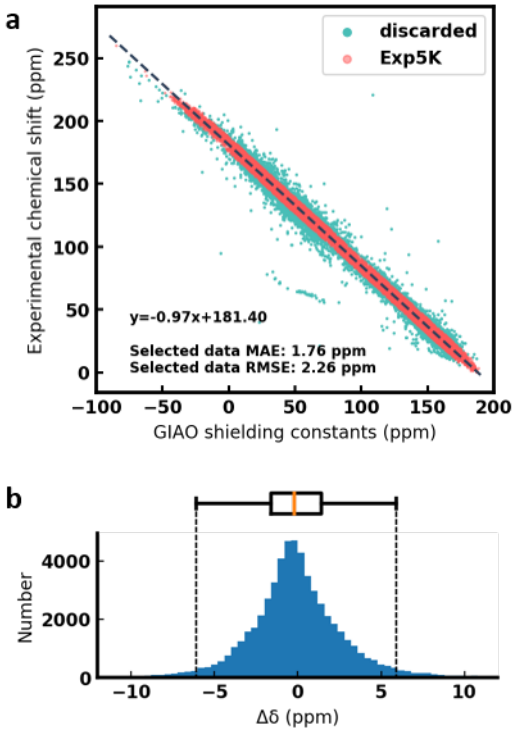

Supplementary Information Figure 6. **(a)** correlation between GIAO DFT shielding constants and experimental chemical shift. **(b)** Histogram shows the error distribution for the correlation. The boxplot shows the lower quartile (Q_1) and upper quartile (Q_3). Whiskers show the minimum (Q_1 – 1.5*IQR) and maximum (Q_3 + 1.5*IQR), which corresponds to -6.1 and 5.9 ppm. Data outside the dash line are considered as outliers due to incorrect assignment of experimental chemical shift, which are discarded, as colored in green in figure **(a)**.

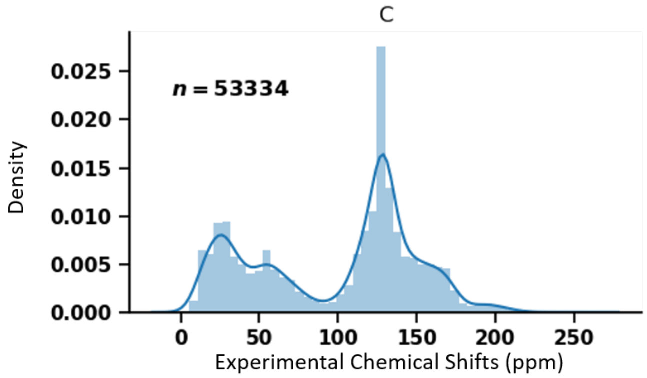

Supplementary Information Figure 7. Distribution of experimental ^{13}C chemical shifts in Exp5K database.

Supplementary Information Text 3 | Learning from experimental chemical shifts. Performance of ExpNN-*dft* trained against experimental chemical shifts using DFT structure inputs are shown in SI Fig. 7.

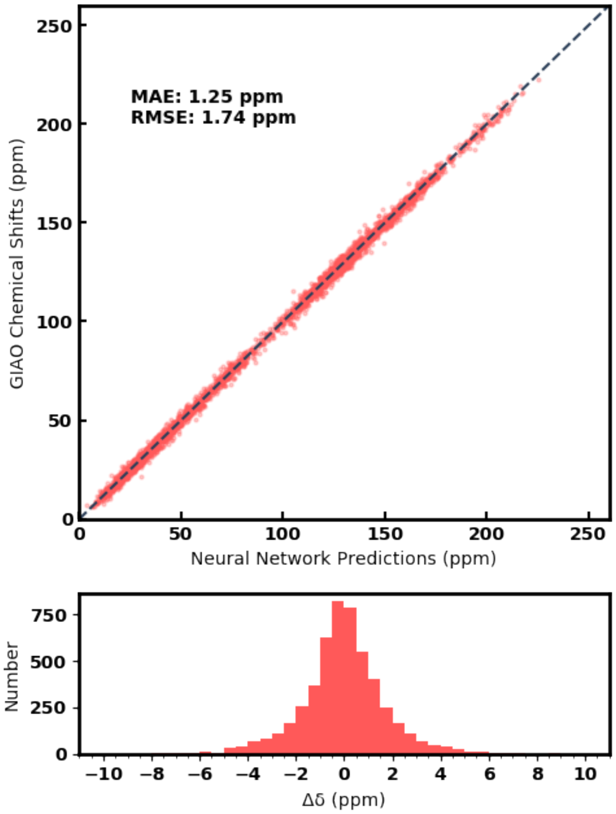

Supplementary Information Figure 8. Scatter plot and histogram show the correlation between chemical shifts predicted from ExpNN-*dft* and observed experimentally for ^{13}C. The grey dash line in the scatter plot indicates a perfect correlation.

Supplementary Information Text 4 | Training on empirical MMFF structures. Performance of ExpNN-*ff* trained against experimental chemical shifts using MMFF structure inputs are shown in SI Fig. 8.

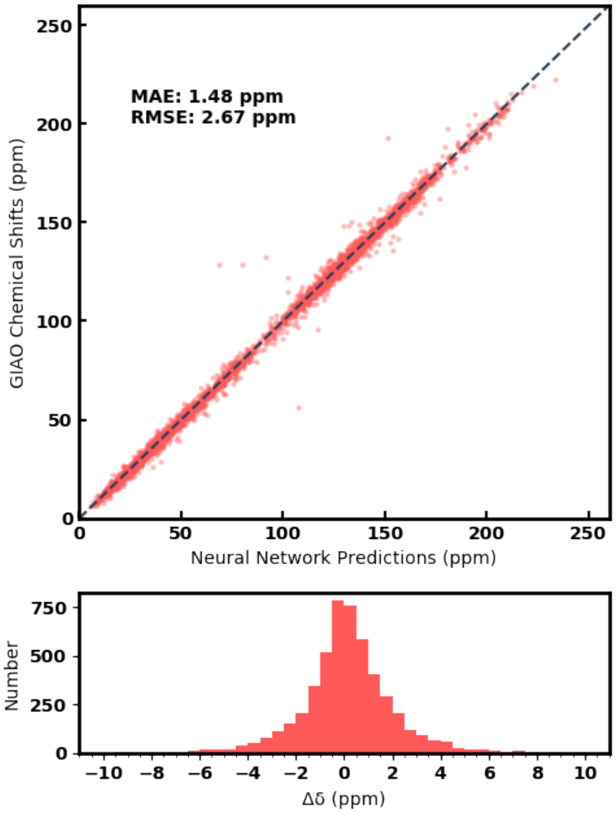

Supplementary Information Figure 9. Scatter plot and histogram show the correlation between chemical shifts predicted from ExpNN-*ff* and observed experimentally for ^{13}C. The grey dash line in the scatter plot indicates a perfect correlation.

Supplementary Information Text 5 | Performance on the CHESHIRE testing set

Performance of three networks discussed in the present work, ExpNN-*ff*, ExpNN-*dft*, and DFTNN are benchmarked against an external validation set, CHESHIRE, which is outside of the NMR8K dataset. The CHESHIRE dataset is composed of small, rigid molecules containingb C, H, N, O and S. We compare these network performances against a fully QM approach (DFT geometry optimization and shielding tensor calculation) and a mixed approach (here termed FFDDT, which involves MMFF geometry optimization and DFT shielding tensor computation). The DFT method selected here is the same used in the generation of the DFT8K database, mPW1PW91/6-311+G(d,p)//M06-2X/def2-TZVP. Molecular structures with atom indices are shown in SI Figure9. Experimental and calculated chemical shifts are shown in SI Table1. We note here that the original CHESHIRE dataset contains 25 neutral molecules, from which one was removed for this study due to the presence of charged atoms in the Lewis structure which are not currently supported by our network.

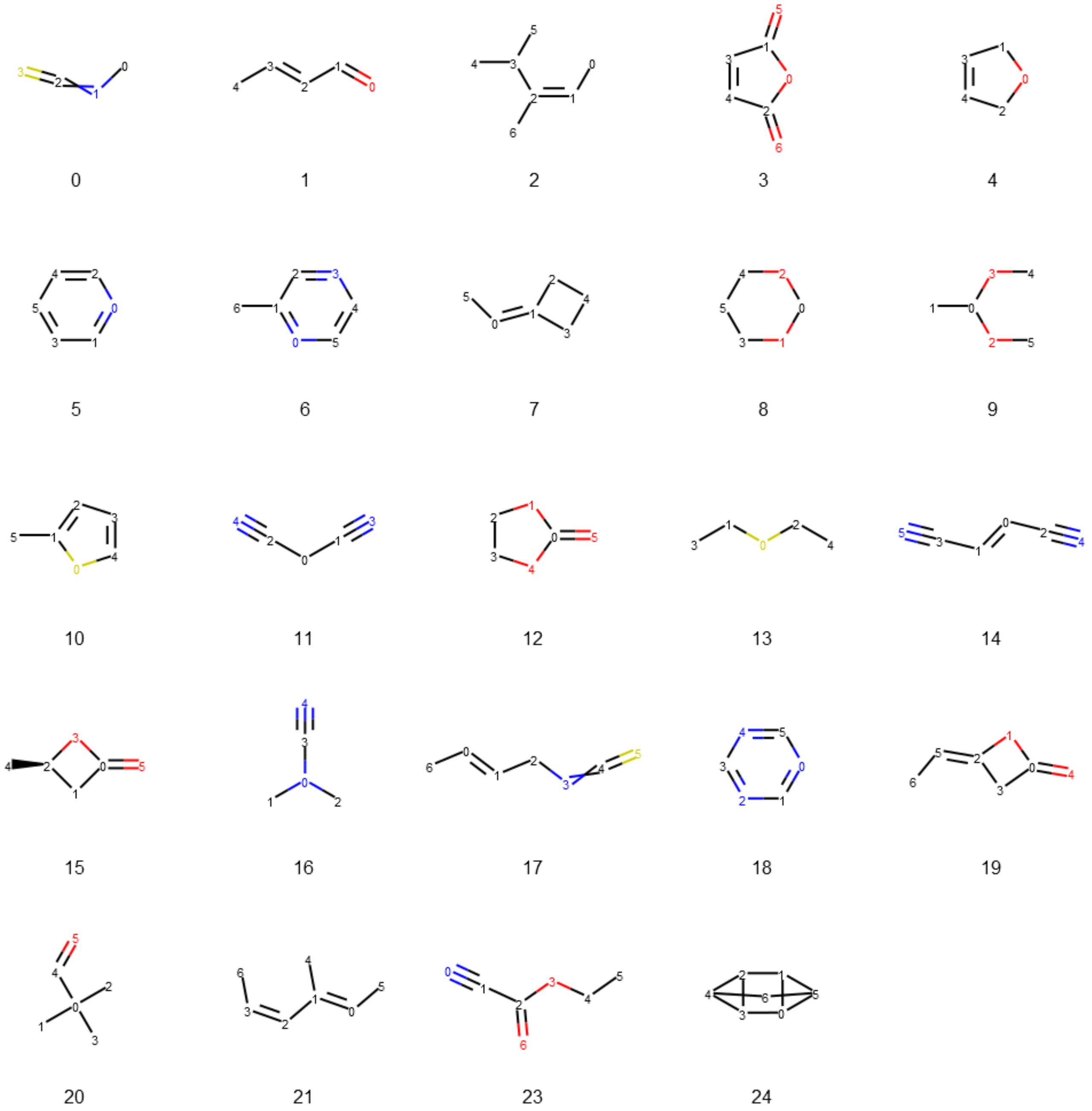

Supplementary Information Figure 10. 24 CHESHIRE molecules tested. The number in legends associated with each molecule is the molecule ID from SI Table1. Numbers in molecules associated with each atom are atom indices from SI Table1.

Supplementary Information Table 1. ^{13}C chemical shifts calculated by neural network and DFT as well as experimentally observed for 24 CHESHIRE molecules. Chemical shifts are in the unit of ppm

mol_id	atom_index	exp[a]	ExpNN-*ff*	ExpNN-*dft*	DFTNN	DFT	FFDFT
0	0	30.48	36.64	30.58	27.07	26.04	34.25
0	2	128.90	126.74	124.53	129.12	123.37	193.04

1	1	194.04	193.98	194.68	191.26	192.26	191.87
1	2	134.61	132.58	132.39	132.39	134.65	133.94
1	3	154.32	152.20	155.02	160.96	160.55	155.05
1	4	18.61	18.06	17.98	19.13	20.31	21.77
2	0	13.21	13.97	13.51	14.21	13.62	14.80
2	1	115.93	118.01	117.24	120.26	119.13	118.64
2	2	141.67	141.69	142.02	144.77	144.72	143.00
2	3	36.99	36.84	37.12	38.99	41.05	40.96
2	4	21.52	21.49	21.28	20.60	21.06	21.60
2	5	21.52	21.37	20.84	19.52	20.00	20.84
2	6	13.06	13.90	13.08	9.26	9.71	10.19
3	1	164.58	163.99	168.63	164.39	164.81	166.26
3	2	164.58	163.99	168.63	164.39	164.81	166.24
3	3	136.76	135.08	136.18	134.87	137.99	133.77
3	4	136.76	135.08	136.18	134.87	137.99	133.76
4	1	75.42	75.95	75.87	75.50	76.07	73.61
4	2	75.42	75.95	75.86	75.49	76.05	73.59
4	3	126.34	125.55	127.57	129.83	128.51	125.84
4	4	126.34	125.56	127.57	129.84	128.52	125.86
5	1	149.94	150.32	150.28	150.83	150.05	148.78
5	2	149.94	150.32	150.27	150.83	150.02	148.76
5	3	123.55	123.76	124.05	123.62	123.51	119.64
5	4	123.55	123.75	124.04	123.61	123.48	119.63
5	5	135.89	136.10	136.32	137.35	136.32	133.31
6	1	154.11	153.80	153.51	154.13	155.23	153.36
6	2	144.85	145.07	144.73	145.67	145.49	144.74
6	4	141.90	142.24	141.39	142.51	141.46	140.39
6	5	143.90	144.52	143.33	143.85	143.97	142.80
6	6	21.53	21.71	21.63	21.91	22.32	22.49
7	0	104.79	106.22	98.41	106.44	102.75	106.13
7	1	150.62	150.79	151.28	159.79	156.05	153.87
7	2	32.08	33.09	33.89	34.86	32.58	29.31
7	3	32.08	33.10	33.89	34.86	32.58	29.32
7	4	16.76	18.61	17.63	19.86	16.96	18.89
8	0	94.28	93.82	93.48	91.44	91.37	91.41
8	3	66.94	66.97	65.89	64.79	65.32	63.95
8	4	66.94	66.97	65.89	64.79	65.32	63.94
8	5	26.64	24.33	24.13	25.44	27.51	28.52
9	0	101.24	102.48	102.03	102.06	101.18	101.88
9	1	18.80	18.87	18.97	16.51	17.29	18.66
9	4	52.31	54.02	53.94	52.02	52.84	52.10
9	5	52.31	54.07	54.11	51.47	51.91	52.27
10	1	139.50	141.72	141.89	144.93	144.20	141.63
10	2	125.14	124.85	125.45	124.75	123.96	121.73
10	3	126.86	126.23	126.49	125.60	125.31	124.19
10	4	123.03	124.98	124.33	127.00	126.04	123.54
10	5	14.95	15.47	15.54	15.32	15.61	16.53
11	0	8.77	6.78	5.19	9.13	7.43	9.48
11	1	109.35	112.77	111.72	114.78	113.71	113.43
11	2	109.35	112.77	111.72	114.78	113.71	113.44
12	0	155.92	154.60	155.45	154.22	153.93	158.22
12	2	65.05	65.21	64.46	64.07	62.97	61.48
12	3	65.05	65.22	64.46	64.07	62.98	61.49

13	1	25.50	24.62	22.57	24.44	23.65	25.51
13	2	25.50	24.63	22.57	24.44	23.66	25.52
13	3	14.80	13.46	13.54	12.09	12.40	14.24
13	4	14.80	13.46	13.53	12.09	12.41	14.23
14	0	119.33	120.53	121.32	120.96	122.93	120.38
14	1	119.33	120.53	121.32	120.96	122.93	120.38
14	2	114.26	114.14	114.61	118.23	117.67	116.19
14	3	114.26	114.14	114.61	118.23	117.67	116.19
15	0	168.39	167.29	168.45	167.14	167.15	168.18
15	1	44.36	39.96	40.06	44.21	44.63	45.96
15	2	68.12	69.95	69.74	68.36	65.59	64.71
15	4	20.58	18.87	19.12	18.65	19.89	20.59
16	1	40.53	39.57	38.92	37.82	38.96	41.14
16	2	40.53	39.57	38.92	37.82	38.96	41.14
16	3	119.36	118.88	120.09	120.99	122.04	116.97
19	0	165.18	163.15	165.86	165.70	165.38	166.68
19	2	147.66	151.54	151.99	154.18	151.38	143.95
19	3	42.40	39.68	38.68	43.01	42.74	45.18
19	5	87.06	91.08	87.91	90.99	86.25	80.39
20	0	42.50	47.52	44.67	42.89	44.31	44.66
20	1	23.44	25.43	22.93	23.56	22.29	22.49
20	2	23.44	25.60	23.44	20.57	20.42	22.44
20	3	23.44	25.43	22.93	23.56	22.30	22.50
20	4	205.83	204.61	205.70	206.29	207.52	209.94
21	0	113.64	115.38	114.52	118.08	117.51	116.60
21	1	142.46	141.65	142.08	145.85	146.18	141.94
21	2	139.82	139.44	139.11	140.90	141.96	137.80
21	3	116.75	113.72	113.10	114.74	114.73	112.73
21	4	17.85	17.97	18.56	17.68	17.99	18.86
23	1	109.62	107.24	108.73	112.05	111.72	109.95
23	2	144.44	147.01	148.17	149.49	143.99	149.32
23	4	65.40	62.47	63.40	63.76	64.36	62.19
23	5	13.73	13.58	13.35	12.62	12.70	14.59
24	0	14.71	18.83	20.02	22.15	14.49	6.98
24	1	14.71	18.83	20.03	22.15	14.50	6.98
24	2	14.71	18.83	20.02	22.15	14.49	6.97
24	3	14.71	18.82	20.02	22.14	14.49	6.96
24	4	22.99	17.31	17.93	24.91	23.79	25.31
24	5	22.99	17.32	17.94	24.91	23.79	25.30
24	6	31.98	27.89	22.46	27.09	32.95	38.26

a: experimental chemical shifts are extracted from the CHESHIRE website[17]

Supplementary Information Table 2. Performance of ^{13}C chemical shift predictions using ExpNN-*ff* compared to 25 electronic structure methods for the CHESHIRE probe set.

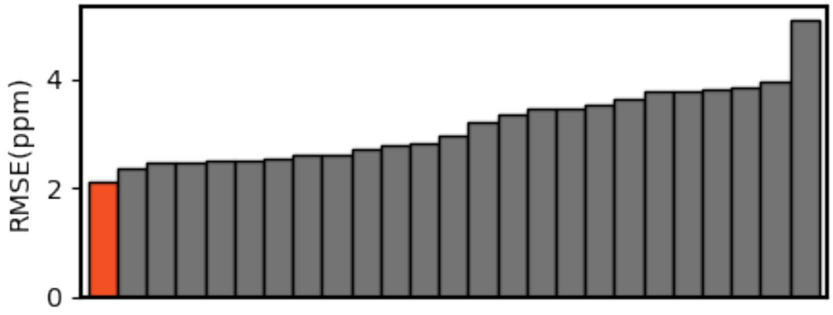

Histogram shows RMSE for the CHESHIRE probe set ^{13}C chemical shift predictions compared with experimental value for ExpNN-*ff* and 25 electronic structure calculation methods. The orange column is the ExpNN-*ff*.

	#	Optimization Method	NMR Calculation Method	RMSD[a]
**Network**	1	MMFF	ExpNN-*ff*	2.12
**Electronic structure method**	2	M062X/6-311+G(2d,p)	mPW1PW91/6-311+G(2d,p)	2.34
	3	M062X/6-31G(d)	mPW1PW91/6-31+G(d,p)	2.47
	4	M062X/6-31+G(d,p)	mPW1PW91/6-311+G(2d,p)	2.47
	5	B3LYP/6-31+G(d,p)	mPW1PW91/6-311+G(2d,p)	2.49
	6	B3LYP/6-31+G(d,p)	PBE0/6-311+G(2d,p)	2.49
	7	M062X/6-31G(d)	mPW1PW91/6-31G(d)	2.53
	8	B3LYP/6-311+G(2d,p)	PBE0/6-311+G(2d,p)	2.59
	9	B3LYP/6-311+G(2d,p)	mPW1PW91/6-311+G(2d,p)	2.59
	10	B3LYP/6-31+G(d,p)	B3LYP/6-311+G(2d,p)	2.71
	11	B3LYP/6-31G(d)	B3LYP/6-31G(d)	2.77
	12	B3LYP/6-311+G(2d,p)	B3LYP/6-311+G(2d,p)	2.80
	13	B3LYP/6-31G(d)	B3LYP/6-31+G(d,p)	2.94
	14	M062X/6-311+G(2d,p)	M06L/6-311+G(2d,p)	3.19
	15	M062X/6-31+G(d,p)	M06L/6-311+G(2d,p)	3.33
	16	B3LYP/6-31+G(d,p)	B3LYP/aug-cc-pVDZ	3.45
	17	M062X/6-31G(d)	M062X/6-31+G(d,p)	3.47
	18	MP2/6-31+G(d,p)	MP2/6-311+G(2d,p)	3.54
	19	M062X/6-31G(d)	M06L/6-31G(d)	3.63
	20	MP2/6-31+G(d,p)	MP2/6-31+G(d,p)	3.77
	21	M062X/6-311+G(2d,p)	M062X/6-311+G(2d,p)	3.79
	22	M062X/6-31+G(d,p)	M062X/6-311+G(2d,p)	3.80
	23	M062X/6-31G(d)	M06L/6-31+G(d,p)	3.84
	24	M062X/6-31G(d)	M062X/6-31G(d)	3.94
	25	B3LYP/6-31+G(d,p)	WC04/aug-cc-pVDZ	5.10
	26	B3LYP/6-31+G(d,p)	VSXC/aug-cc-pVDZ	7.59

a: RMSE for electronic structure methods are from the CHESHIRE website

Supplementary Information Text 6 | Quantitative comparison for three examples involving stereochemical and conformational factors in Scheme 1. (^{13}C NMR chemical shifts)

a. diastereomers

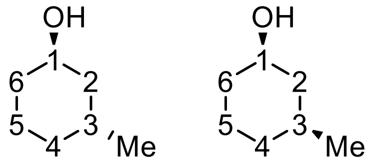

cis *trans*

	exp cis[18]	NN cis	error	NN trans	error	exp trans[18]	NN cis	error	NN trans	error
C1	70.8	68.8	2.0	68.0	2.8	67.1	68.8	1.7	68.0	0.9
C2	44.7	43.8	0.9	43.7	1.0	41.7	43.8	2.06	43.7	2.0
C3	31.6	30.9	0.7	28.6	3.0	26.7	30.9	4.24	28.6	1.9
C4	34.2	33.9	0.3	34.6	0.4	34.3	33.9	0.41	34.6	0.3
C5	24.3	23.6	0.7	21.6	2.7	20.2	23.6	3.43	21.6	1.4
C6	35.3	34.6	0.7	34.4	0.9	33.2	34.6	1.36	34.4	1.2
Me	22.5	21.9	0.6	22.0	0.5	22.2	21.9	0.29	22.0	0.2
MAE			0.8		1.6			1.9		1.1

The cis- and trans-diastereomers of 1,3-hydroxymethylcyclohexane are distinguishable by our network. Assignment of the diastereomers based on the predicted ^{13}C chemical shifts is correct.

b. diastereotopic groups

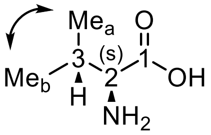

	exp[a]	NN predicted
C1	175.08	173.92
C2	61.52	60.74
C3	30.12	31.00
Me$_a$	17.77	17.64
Me$_b$	19.07	18.54

a: experimental data extracted from ChemicalBook: (https://www.chemicalbook.com/SpectrumEN_72-18-4_13CNMR.htm

Diastereotopic methyl groups in L-valine are differentiated by the network. The predicted $\Delta\delta$ value is 0.9 ppm, while experimentally it is 1.3 ppm.

c. conformers

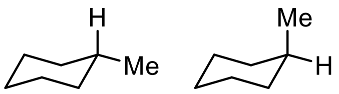

$conf_a$ $conf_b$

	Exp[18]	$conf_a$	$conf_b$	average
C1	33.1	32.8	32.6	32.7
C2	35.8	34.9	34.5	34.9
C3	26.6	26.2	25.2	26.1
C4	26.3	26.9	26.7	26.9
C5	26.6	26.2	25.2	26.1
C6	35.8	34.9	34.5	34.9
Me	22.7	21.2	19.4	21.1

The ring-flipped chair forms of methylcyclohexane are differentiated by our network. The two conformers provide different ^{13}C predictions, with the largest effect evident at the methyl group, which is oriented equatorially or axially in the two conformers. The final prediction is based on a Boltzmann weighting over all conformers, and compares favorably with experiment.

Supplementary Information Text 7 | Structural assignment Chemical structure and atom indices as well as experimental and predicted ^{13}C chemical shifts for six structures in Figure 6 are shown here.

a.

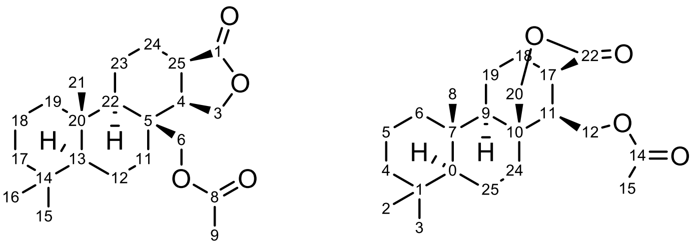

Original Revised

		Original			Revised		
#[a]	exp[19]	atom_index	predicted	error[b]	atom_index	predicted	error[b]
1	173.57	1	177.85	4.3	22	174.50	0.9
2	170.85	8	169.32	1.5	14	169.95	0.9
3	74.85	3	65.47	9.4	20	67.91	6.9
4	62.68	6	64.82	2.1	12	62.84	0.2
5	58.82	13	56.99	1.8	9	59.06	0.2
6	56.32	22	56.80	0.5	0	57.84	1.5
7	48.63	4	49.42	0.8	11	51.01	2.4
8	41.63	25	42.70	1.1	4	42.09	0.5
9	40.80	17	42.20	1.4	6	41.07	0.3
10	39.82	19	41.86	2.0	17	40.19	0.4
11	37.95	5	41.72	3.8	7	37.43	0.5
12	37.18	11	36.97	0.2	10	36.79	0.4
13	35.73	20	36.85	1.1	24	35.38	0.4
14	33.19	14	34.85	1.7	1	34.93	1.7
15	33.19	15	31.35	1.8	2	31.94	1.3
16	31.04	24	23.46	7.6	18	31.58	0.5
17	21.43	16	23.31	1.9	3	23.58	2.2
18	20.80	9	20.81	0.0	19	21.58	0.8
19	19.74	12	19.21	0.5	15	20.73	1.0
20	18.70	23	19.02	0.3	25	20.27	1.6
21	18.33	18	18.97	0.6	5	18.78	0.5
22	15.88	21	15.21	0.7	8	16.75	0.9

a: Orders corresponding to the error bar plot below.
b: Absolute error

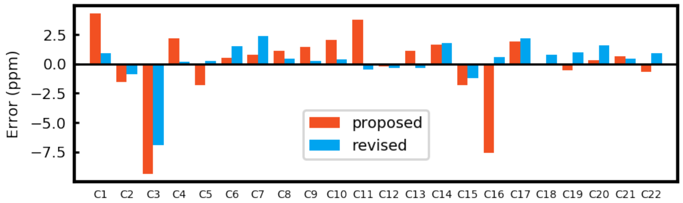

b.

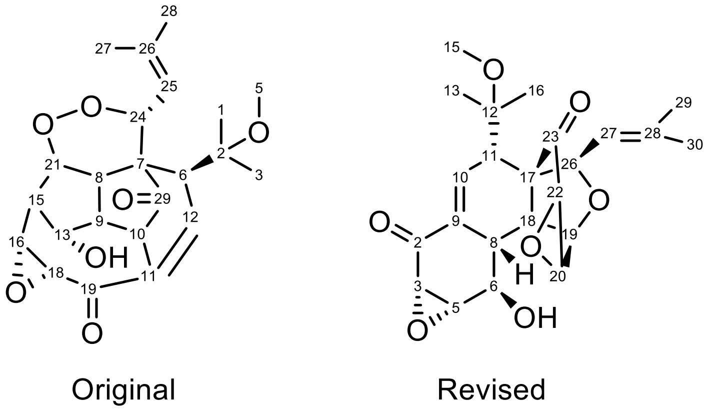

#	exp[20]	Original atom index	Original predicted	Original error	Revised atom index	Revised predicted	Revised error
1	202.9	28	209.2	6.3	22	199.3	3.6
2	192.8	18	194.1	1.3	1	191.0	1.8
3	142.2	25	140.7	1.5	9	144.5	2.3
4	139.6	11	138.8	0.8	27	135.9	3.7
5	132.5	10	138.1	5.6	8	134.9	2.4
6	120.7	24	118.9	1.8	26	120.6	0.1
7	77.3	20	83.5	6.2	11	79.4	2.1
8	75.8	23	83.3	7.5	25	78.1	2.3
9	72.7	1	78.3	5.6	18	76.9	4.2
10	71.5	12	70.3	1.2	5	74.0	2.5
11	61	17	61.8	0.8	2	60.8	0.2
12	60.5	6	59.0	1.5	4	58.3	2.2
13	54.5	7	58.6	4.1	16	57.0	2.5
14	53.2	15	57.9	4.7	21	53.7	0.5
15	53.1	5	53.4	0.3	19	52.8	0.3
16	49.1	4	51.5	2.4	14	50.0	0.9
17	47.8	9	49.4	1.6	10	48.3	0.5
18	40.9	14	48.3	7.4	17	46.3	5.4
19	40.4	8	45.3	4.9	7	39.2	1.2
20	26.6	0	28.9	2.3	15	24.9	1.7
21	26.1	2	25.2	0.9	12	24.9	1.2
22	24.7	26	23.1	1.6	29	22.8	1.9
23	18.6	27	23.0	4.4	28	22.8	4.2

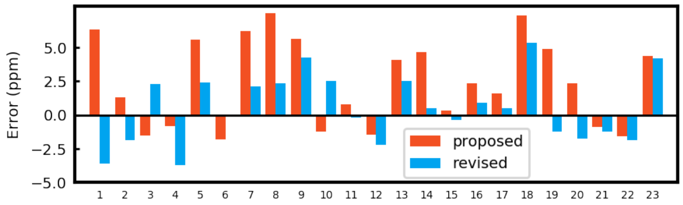

c.

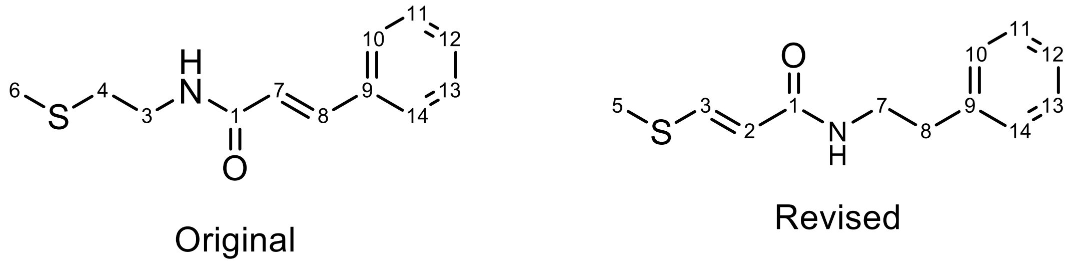

#	exp[21]	Original atom index	Original predicted	Original error	Revised atom index	Revised predicted	Revised error
1	167.3	1	163.8	3.5	1	164.5	2.8
2	143.5	3	144.9	1.4	8	141.1	2.4
3	140.5	9	139.2	1.3	9	135.1	5.4
4	129.8	11	128.9	0.9	12	129.4	0.4
5	129.8	13	128.9	0.9	13	128.6	1.2
6	129.5	14	128.3	1.2	11	128.6	0.9
7	129.5	10	128.2	1.3	10	128.3	1.2
8	127.3	12	126.4	0.9	14	128.0	0.7
9	116.6	2	117.1	0.5	7	119.8	3.2
10	42.0	7	40.4	1.6	3	39.9	2.1
11	36.6	8	35.5	1.1	4	33.6	3.0
12	14.3	5	16.1	1.8	6	15.6	1.3

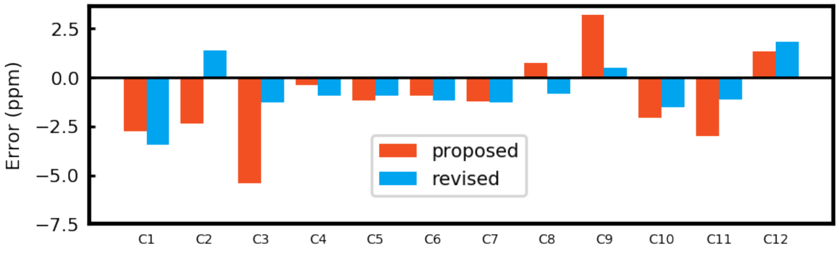

d.

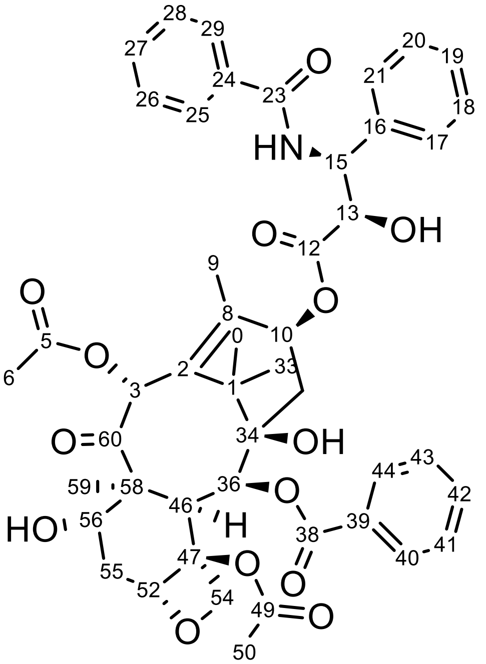

Incorrect

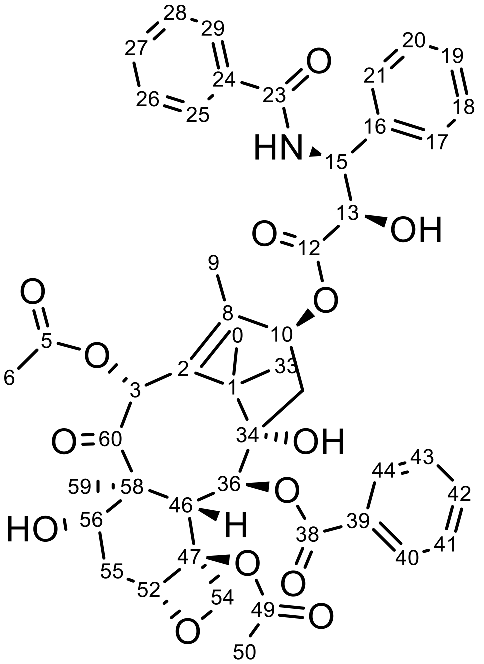

correct

		Incorrect			Revised		
#	exp[22]	atom index	predicted	error	atom index	predicted	error
1	203.6	60	210.2	6.6	60	206.1	2.5
2	172.7	12	171.5	1.2	12	171.6	1.1
3	171.2	49	169.3	1.9	5	170.1	1.1
4	170.4	5	168.6	1.8	38	167.3	3.1
5	167.0	38	165.8	1.2	49	167	0
6	167.0	23	165.6	1.4	23	165.8	1.2
7	142.0	16	139.9	2.1	2	140.7	1.3
8	138.0	2	138.4	0.4	16	139	1
9	133.7	8	137.9	4.2	8	134	0.3
10	133.6	24	132.7	0.9	24	133.1	0.5
11	133.2	42	132.6	0.6	42	133	0.2
12	131.9	27	131.3	0.6	27	131.6	0.3
13	130.2	39	130.5	0.3	44	130.2	0
14	130.2	40	130.0	0.2	39	129.4	0.8
15	129.1	20	129.1	0.0	40	129	0.1
16	129.0	44	128.8	0.2	20	128.6	0.4
17	129.0	43	128.6	0.4	18	128.5	0.5
18	128.7	28	128.4	0.3	28	128.4	0.3
19	128.7	18	128.4	0.3	26	128.4	0.3
20	128.7	41	128.4	0.3	41	128.2	0.5
21	128.7	26	128.2	0.4	29	127.9	0.8
22	128.3	29	128.1	0.2	43	127.8	0.5
23	127.0	19	127.8	0.8	17	127.7	0.7
24	127.0	25	127.8	0.8	25	127.4	0.4
25	127.0	17	127.4	0.4	21	127.4	0.4

26	127.0	21	127.2	0.2	19	127.3	0.3
27	84.4	52	82.3	2.1	47	80.9	3.5
28	81.1	47	81.5	0.4	34	79.5	1.6
29	79.0	34	79.7	0.7	52	76.7	2.3
30	76.5	10	77.9	1.4	36	76.2	0.3
31	75.5	3	75.4	0.1	3	75.1	0.4
32	74.9	13	73.2	1.7	54	73.6	1.3
33	73.2	36	72.4	0.8	10	73.5	0.3
34	72.3	54	71.2	1.1	13	72.3	0
35	72.2	56	70.8	1.4	56	72	0.2
36	58.6	58	56.7	1.9	15	57.5	1.1
37	55.0	15	56.6	1.6	58	53.7	1.3
38	45.6	1	44.6	1.0	46	44.5	1.1
39	43.2	46	44.5	1.3	1	42.9	0.3
40	35.7	32	36.6	0.9	32	33.2	2.5
41	35.6	55	30.8	4.8	55	32.4	3.2
42	26.9	33	24.2	2.7	0	24.6	2.3
43	22.6	6	21.1	1.5	33	24.4	1.8
44	21.8	59	20.8	1.0	6	20.8	1
45	20.8	50	19.4	1.4	50	19.9	0.9
46	14.8	0	19.4	4.6	9	12.8	2
47	9.5	9	15.0	5.5	59	11.9	2.4

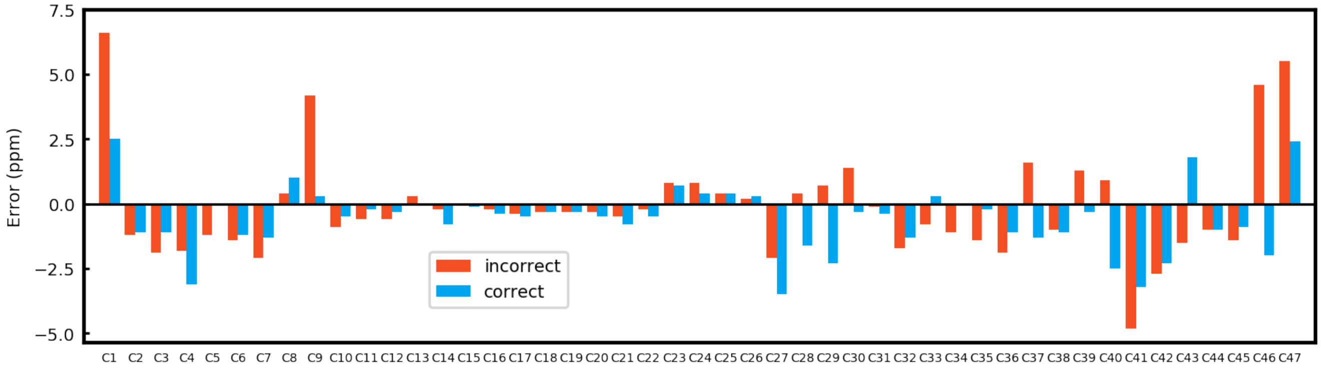

e.

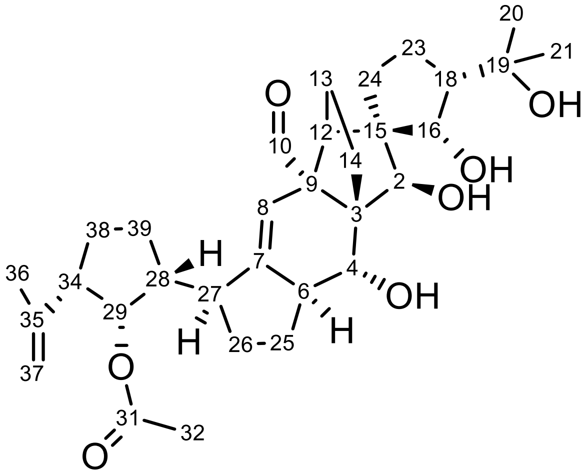

Proposed

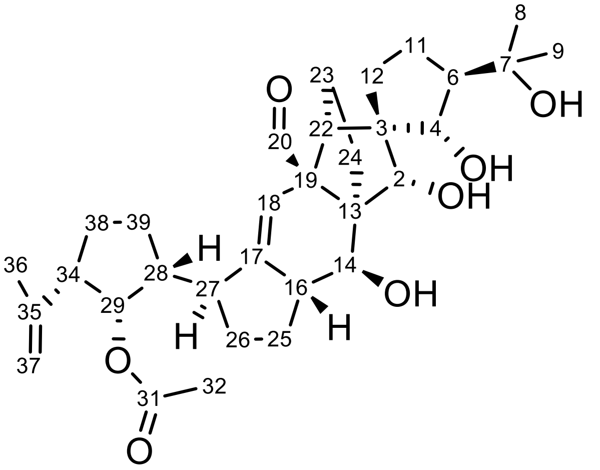

Revised

#	exp[23]	Proposed atom index	Proposed predicted	error	Revised atom index	Revised predicted	error
1	201.91	10	204.78	2.87	20	205.30	3.39
2	172.11	31	169.61	2.50	31	169.75	2.36
3	157.40	7	154.41	2.99	17	152.20	5.20
4	144.79	35	145.22	0.44	35	144.43	0.36
5	114.76	8	120.81	6.05	18	122.07	7.30
6	111.44	37	109.68	1.76	37	107.79	3.65
7	78.07	16	79.88	1.81	29	77.93	0.14
8	76.91	29	79.68	2.76	2	77.13	0.22
9	75.69	2	77.38	1.68	4	74.56	1.13
10	72.17	4	74.48	2.31	19	74.52	2.35
11	71.17	9	74.44	3.27	14	74.50	3.33
12	68.84	19	72.58	3.74	7	72.47	3.62
13	62.52	15	55.40	7.11	6	59.37	3.15
14	58.57	3	54.47	4.10	13	58.26	0.31
15	57.88	6	53.50	4.38	3	56.62	1.26
16	52.67	18	52.23	0.44	16	52.08	0.58
17	52.13	34	50.43	1.69	34	51.91	0.22
18	51.44	12	49.21	2.23	28	50.17	1.28
19	50.74	28	47.42	3.32	22	50.12	0.62
20	45.22	27	43.48	1.74	27	43.83	1.39
21	39.04	24	32.74	6.30	12	34.81	4.23
22	33.66	39	31.04	2.62	25	30.28	3.38
23	31.01	26	30.29	0.72	26	30.08	0.94
24	29.88	20	30.26	0.37	39	29.41	0.47
25	29.41	25	30.01	0.60	8	28.38	1.04
26	29.00	21	28.53	0.46	24	28.21	0.79
27	26.63	14	28.15	1.52	9	27.94	1.31
28	24.49	38	27.48	2.99	38	26.72	2.23
29	24.37	13	25.09	0.72	23	25.89	1.52
30	24.01	23	23.71	0.30	11	25.57	1.56
31	23.25	36	22.56	0.68	36	23.73	0.49

32	20.95	32	21.13	0.18	32	21.64	0.69

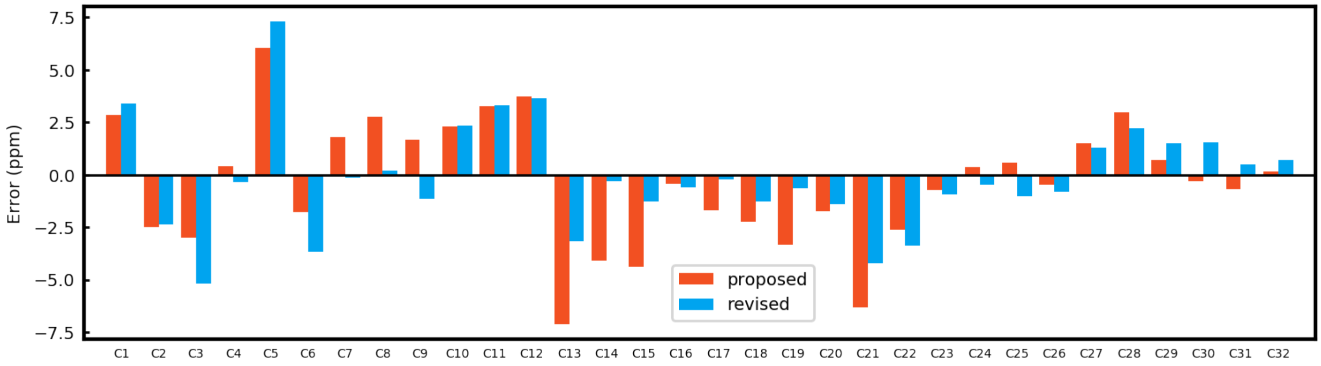

f. source of experimental chemical shift.[24]

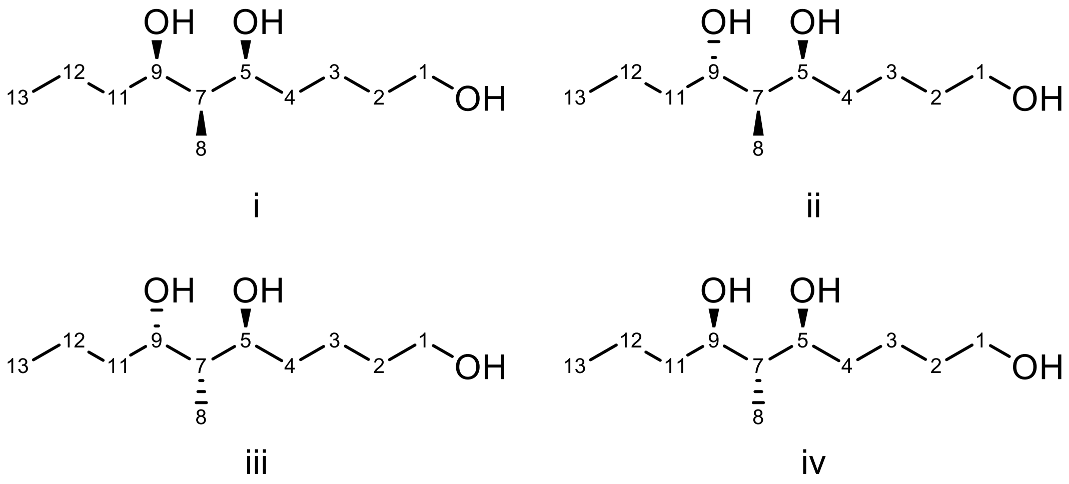

Comparison between ExpNN-ff predictions for i-iv and experimental ^{13}C chemical shift for i

	exp	i	i_error	ii	ii_error	iii	iii_error	iv	iv_error
1	4.40	7.88	3.48	12.79	8.39	12.50	8.10	12.04	7.64
2	14.16	14.66	0.50	14.33	0.17	14.48	0.32	14.28	0.12
3	19.26	19.46	0.19	19.11	0.15	19.75	0.49	18.48	0.79
4	22.46	22.82	0.36	23.10	0.64	24.02	1.56	21.53	0.93
5	32.41	33.04	0.63	32.94	0.53	31.46	0.95	32.19	0.22
7	34.89	35.57	0.68	34.45	0.44	32.76	2.12	33.21	1.68
8	37.44	37.88	0.45	38.07	0.63	36.69	0.75	36.67	0.77
9	40.45	44.35	3.91	42.43	1.99	44.30	3.85	43.88	3.44
11	62.39	62.67	0.28	62.88	0.49	62.24	0.15	62.14	0.25
12	76.96	73.86	3.09	71.73	5.22	73.06	3.89	73.68	3.28
13	77.12	74.57	2.55	73.85	3.27	73.93	3.19	75.04	2.07

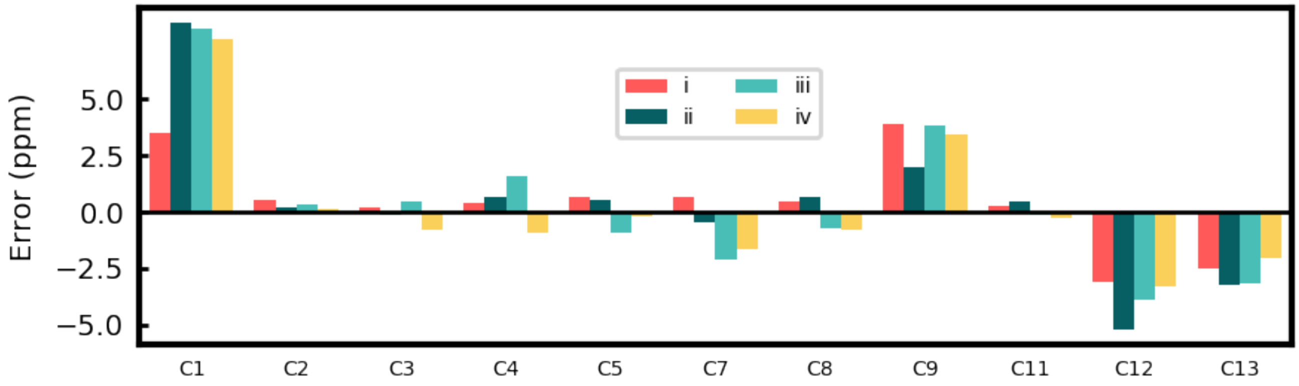

Comparison between ExpNN-ff predictions for i-iv and experimental ^{13}C chemical shift for ii

	exp	i	i_error	ii	ii_error	iii	iii_error	iv	iv_error
1	11.66	7.88	3.79	12.79	1.13	12.50	0.84	12.04	0.38
2	14.21	14.66	0.46	14.33	0.13	14.48	0.28	14.28	0.08
3	19.03	19.46	0.43	19.11	0.08	19.75	0.72	18.48	0.55
4	22.63	22.82	0.19	23.10	0.47	24.02	1.39	21.53	1.10
5	32.58	33.04	0.46	32.94	0.36	31.46	1.12	32.19	0.39
7	33.60	35.57	1.97	34.45	0.85	32.76	0.83	33.21	0.39
8	37.81	37.88	0.08	38.07	0.26	36.69	1.12	36.67	1.14
9	41.38	44.35	2.97	42.43	1.05	44.30	2.92	43.88	2.50
11	62.69	62.67	0.02	62.88	0.19	62.24	0.45	62.14	0.55
12	72.49	73.86	1.37	71.73	0.76	73.06	0.57	73.68	1.19
13	75.83	74.57	1.26	73.85	1.98	73.93	1.90	75.04	0.78

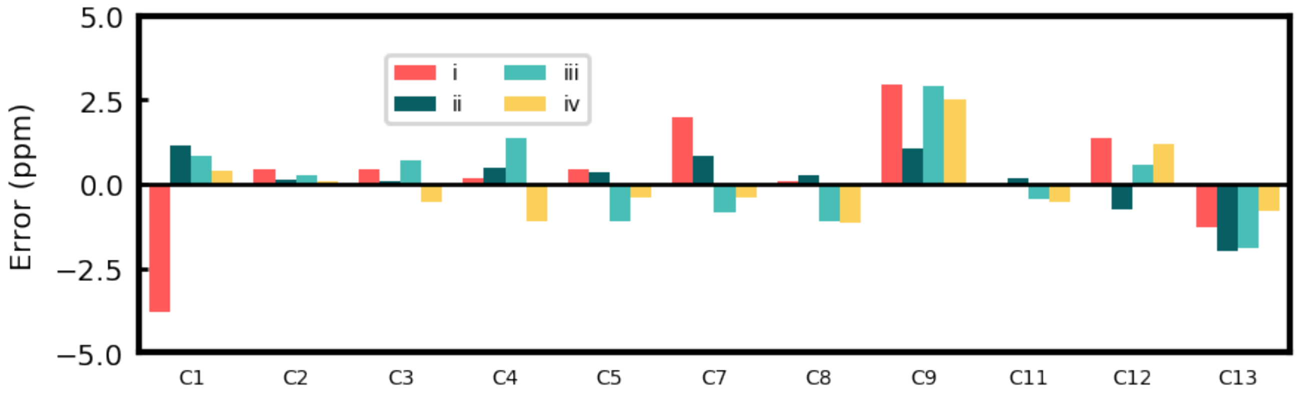

Comparison between ExpNN-ff predictions for i-iv and experimental ^{13}C chemical shift for iii

	exp	i	i_error	ii	ii_error	iii	iii_error	iv	iv_error
1	11.66	7.88	3.79	12.79	1.13	12.50	0.84	12.04	0.38
2	14.21	14.66	0.46	14.33	0.13	14.48	0.28	14.28	0.08
3	19.03	19.46	0.43	19.11	0.08	19.75	0.72	18.48	0.55
4	22.63	22.82	0.19	23.10	0.47	24.02	1.39	21.53	1.10
5	32.58	33.04	0.46	32.94	0.36	31.46	1.12	32.19	0.39
7	33.60	35.57	1.97	34.45	0.85	32.76	0.83	33.21	0.39
8	37.81	37.88	0.08	38.07	0.26	36.69	1.12	36.67	1.14
9	41.38	44.35	2.97	42.43	1.05	44.30	2.92	43.88	2.50
11	62.69	62.67	0.02	62.88	0.19	62.24	0.45	62.14	0.55
12	72.49	73.86	1.37	71.73	0.76	73.06	0.57	73.68	1.19
13	75.83	74.57	1.26	73.85	1.98	73.93	1.90	75.04	0.78

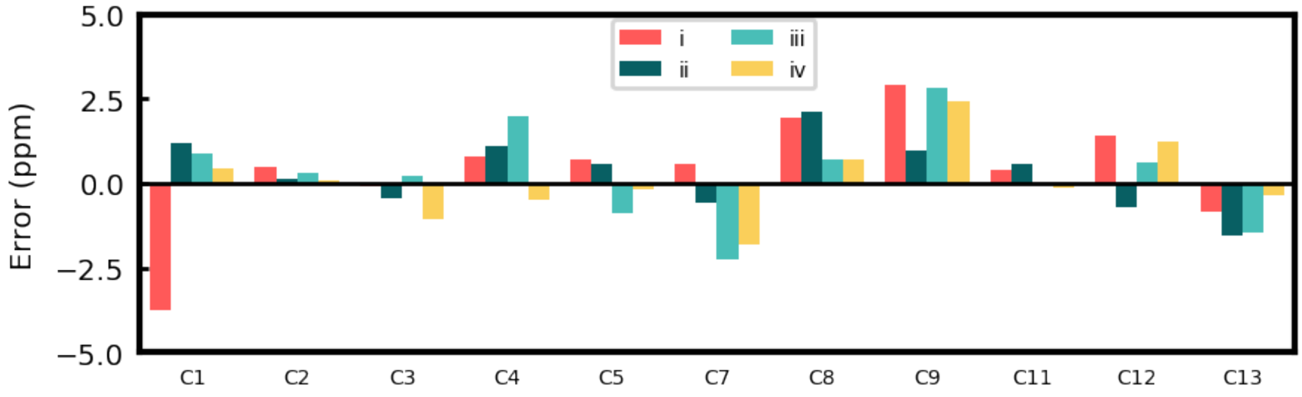

Comparison between ExpNN-ff predictions for i-iv and experimental ^{13}C chemical shift for iv

	exp	i	i_error	ii	ii_error	iii	iii_error	iv	iv_error
1	12.88	7.88	5.00	12.79	0.08	12.50	0.38	12.04	0.84
2	14.23	14.66	0.43	14.33	0.10	14.48	0.25	14.28	0.05
3	18.26	19.46	1.20	19.11	0.85	19.75	1.49	18.48	0.22
4	21.40	22.82	1.42	23.10	1.70	24.02	2.61	21.53	0.13
5	32.29	33.04	0.75	32.94	0.65	31.46	0.84	32.19	0.11
7	33.96	35.57	1.61	34.45	0.48	32.76	1.20	33.21	0.76
8	36.98	37.88	0.91	38.07	1.09	36.69	0.29	36.67	0.31
9	43.68	44.35	0.67	42.43	1.25	44.30	0.62	43.88	0.20
11	62.07	62.67	0.60	62.88	0.81	62.24	0.17	62.14	0.07
12	75.87	73.86	2.01	71.73	4.14	73.06	2.81	73.68	2.19
13	75.89	74.57	1.32	73.85	2.05	73.93	1.96	75.04	0.85

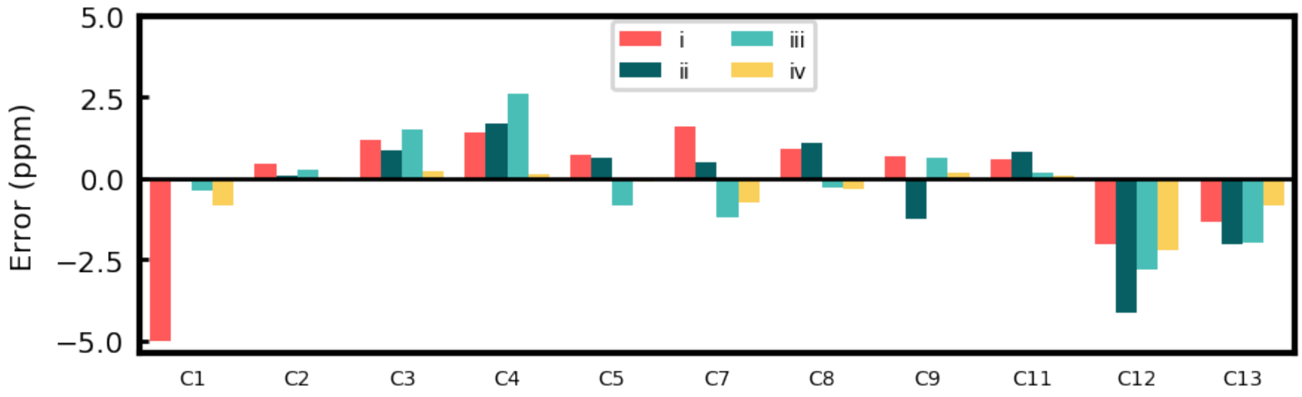

Supplementary Information Text 8 | Conformer based predictions for flexible molecules.

For the above cases **a-f**, the prediction is based on a conformational ensemble. For each molecule, 200 conformers were embedded through distance geometry using RDKit Those conformers are optimized with the Merck Molecular Force Field (MMFF). An energy threshold of 10 kcal/mol is applied to filter relatively stable conformers. A root-mean-square deviation (RMSD) of 0.5 is applied to remove repeated conformers. The coordinates for each low energy conformer are processed by the neural network to predict the conformer specific chemical shift. The final prediction δ is the Boltzmann weighted chemical shift across all conformers through equation 5:

$$\delta = \frac{e^{-E_i/kT}\delta_i}{\sum_i e^{-E_i/kT}}$$

where E_i is the relative energy with respect to the lowest lying conformer calculated through MMFF; δ_i is the predicted chemical shift for conformer i; k is the Boltzmann constant; And T is the room temperature.

Here we show that our model does indeed provide different predictions for individual conformers. For example, for case **f** shown above, the relative predicted chemical shift (Δδ) values differ by up to 10 ppm depending on the carbon atom in question. It is noticeable that the terminal positions (atoms 1 and 13) show smaller variation than the central carbons for this flexible structure. The Boltzmann weighted chemical shift is shown in blue in SI figure 10a.

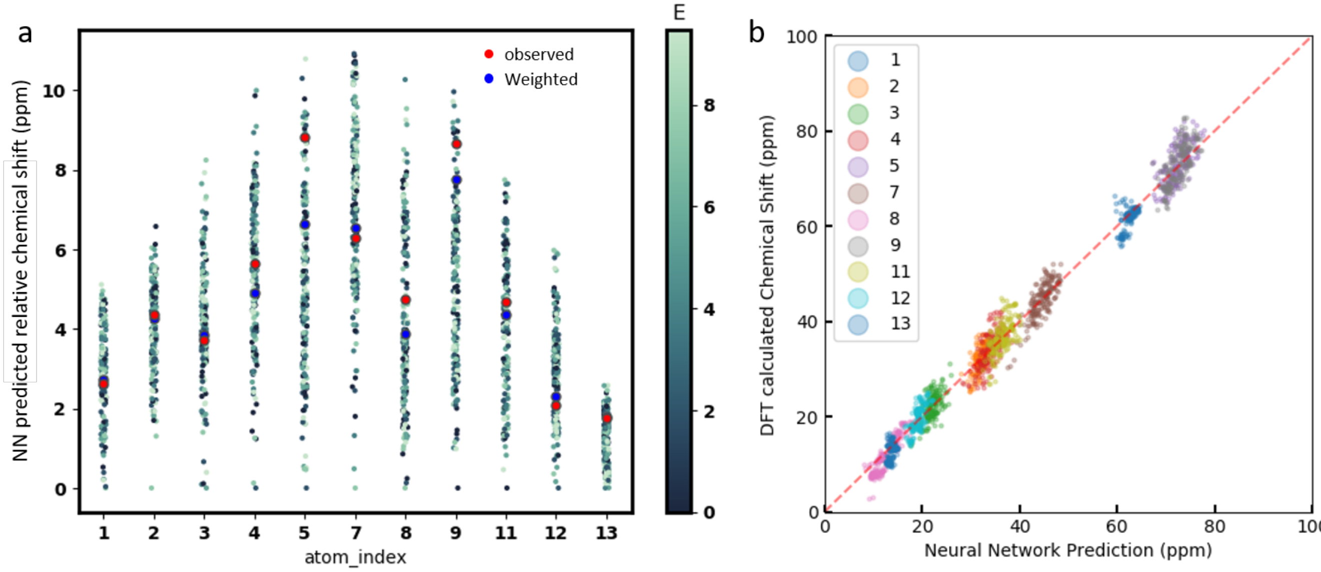

Supplementary Information Figure 11 | Conformer based chemical shift predictions for case f-iv. a. Neural network predicted chemical shifts for all C atoms across 177 conformers. Chemical shifts for each atom are relative chemical shift with respect to the lowest chemical shift predicted for that atom. Each green dot represents a single conformer. The color indicates the relative energy of each conformer, as depicted in the color bar to the right. The large red dot is the experimental observed chemical shift for that carbon atom, which reflect an ensemble of molecular conformations. The Boltzmann weighted predicted chemical shift are shown as large blue dot for each atom. **b.** correlation between neural network predicted chemical shift and DFT calculated chemical shift for each conformer. Different color indicates different C atoms. Each dot represents a single conformer.

Supplementary Information Text 9 | High-throughput detection and revision of incorrectly assigned chemical shifts. Using ExpNN-*ff*, we predicted ^{13}C NMR chemical shift for molecules with MW > 500 in NMRShiftDB. For each molecule, a conformer search was carried out. We then obtained 37,476 unique conformers across 555 molecules. For each molecule, ^{13}C chemical shifts were predicted based on Boltzmann weighting over all conformers. The MAE between predicted and assigned experimental ^{13}C chemical shifts was calculated for each molecule. Molecules with MAE values > 3.5 ppm were considered as candidates for reassignments. We did this in a naïve fashion, comparing the ordered lists of NN predictions and experimental values to inspect the effect that this reassignment would have on the MAE value. Large reductions in MAE values are indicative of problems in the original assignment (e.g. molecule 20244176, which experiences a reduction in MAE by more than 50 ppm). The MAE values before and after reassignment are listed in SI table 3, along with the corresponding ID found in the NMRShiftDB for each molecule.

	ID[a]	Spectrum[b]	Original MAE[c]	Revised MAE[d]
0	2286	Spectrum 13C 0	3.54	2.76
1	2296	Spectrum 13C 0	4.86	4.64
2	2534	Spectrum 13C 0	3.55	2.50
3	2597	Spectrum 13C 0	6.06	4.70
4	2597	Spectrum 13C 1	6.06	4.70
5	2604	Spectrum 13C 0	3.53	3.53
6	2819	Spectrum 13C 0	4.71	2.58
7	3160	Spectrum 13C 0	3.72	2.12
8	3913	Spectrum 13C 0	3.56	2.84
9	4207	Spectrum 13C 0	3.56	3.38
10	7188	Spectrum 13C 0	3.62	2.64
11	7195	Spectrum 13C 0	3.69	2.89
12	10043	Spectrum 13C 0	3.77	3.46
13	11387	Spectrum 13C 0	6.01	2.74
14	21827	Spectrum 13C 0	3.50	3.00
15	21827	Spectrum 13C 1	3.50	3.00
16	22207	Spectrum 13C 0	4.22	3.61
17	75795	Spectrum 13C 0	5.96	3.20
18	10008979	Spectrum 13C 0	3.73	2.89
19	10021719	Spectrum 13C 0	3.63	1.62
20	10022062	Spectrum 13C 0	5.07	3.78
21	10027686	Spectrum 13C 0	3.81	2.94
22	10028028	Spectrum 13C 0	4.53	3.25
23	20000340	Spectrum 13C 0	3.59	2.58
24	20025052	Spectrum 13C 0	3.91	2.31
25	20052875	Spectrum 13C 0	15.57	15.57
26	20101468	Spectrum 13C 0	4.51	1.27
27	20105211	Spectrum 13C 0	3.71	3.36
28	20131575	Spectrum 13C 0	3.83	2.55
29	20209747	Spectrum 13C 0	4.02	2.44
30	20213329	Spectrum 13C 0	5.65	3.45
31	20244176	Spectrum 13C 0	58.00	1.68
32	20244313	Spectrum 13C 0	21.60	20.44
33	20253108	Spectrum 13C 0	4.11	2.72
34	30079971	Spectrum 13C 0	3.57	2.09
35	30080366	Spectrum 13C 0	3.66	2.30
36	30081834	Spectrum 13C 0	4.56	3.85
37	30082581	Spectrum 13C 0	3.69	3.47
38	30082583	Spectrum 13C 0	4.12	3.78
39	30082584	Spectrum 13C 0	6.16	6.13
40	40097659	Spectrum 13C 0	4.30	1.82

41	40115986	Spectrum 13C 0	4.92	3.29
42	60002036	Spectrum 13C 0	4.72	4.04

a: Structure ID found in NMRShiftDB
b: Name for each spectrum found in NMRShiftDB
c,d: MAE for ^{13}C chemical shift in the unit of ppm

References

1 Schütt, K. T., Arbabzadah, F., Chmiela, S., Müller, K. R. & Tkatchenko, A. Quantum-chemical insights from deep tensor neural networks. *Nature communications* **8**, 13890 (2017).

2 Jørgensen, P. B., Jacobsen, K. W. & Schmidt, M. N. Neural Message Passing with Edge Updates for Predicting Properties of Molecules and Materials. *arXiv preprint arXiv:1806.03146* (2018).

3 Schütt, K. T., Sauceda, H. E., Kindermans, P.-J., Tkatchenko, A. & Müller, K.-R. SchNet–A deep learning architecture for molecules and materials. *The Journal of Chemical Physics* **148**, 241722 (2018).

4 Chollet, Franc¸ois. Keras: Deep learning library for theano and tensorflow. https://github.com/fchollet/keras, 2015.

5 Abadi, M. *et al.* in *12th {USENIX} Symposium on Operating Systems Design and Implementation ({OSDI} 16).* 265-283.

6 Pedregosa, F. *et al.* Scikit-learn: Machine learning in Python. *Journal of machine learning research* **12**, 2825-2830 (2011).

7 RDKit: Open-source cheminformatics; http://www.rdkit.org

8 Kingma, D. P. & Ba, J. Adam: A method for stochastic optimization. *arXiv preprint arXiv:1412.6980* (2014).

9 Steinbeck, C., Krause, S. & Kuhn, S. NMRShiftDB constructing a free chemical information system with open-source components. *Journal of chemical information and computer sciences* **43**, 1733-1739 (2003).

10 Ceriotti, M., Tribello, G. A. & Parrinello, M. Demonstrating the transferability and the descriptive power of sketch-map. *Journal of chemical theory and computation* **9**, 1521-1532 (2013).

11 Nikolova, N. & Jaworska, J. Approaches to measure chemical similarity–a review. *QSAR & Combinatorial Science* **22**, 1006-1026 (2003).

12 Halgren, T. A. Merck molecular force field. I. Basis, form, scope, parameterization, and performance of MMFF94. *Journal of computational chemistry* **17**, 490-519 (1996).

13 Gaussian 16, Revision B.01, Frisch, M. J.; Trucks, G. W.; Schlegel, H. B.; Scuseria, G. E.; Robb, M. A.; Cheeseman, J. R.; Scalmani, G.; Barone, V.; Petersson, G. A.; Nakatsuji, H.; Li, X.; Caricato, M.; Marenich, A. V.; Bloino, J.; Janesko, B. G.; Gomperts, R.; Mennucci, B.; Hratchian, H. P.; Ortiz, J. V.; Izmaylov, A. F.; Sonnenberg, J. L.; Williams-Young, D.; Ding, F.; Lipparini, F.; Egidi, F.; Goings, J.; Peng, B.; Petrone, A.; Henderson, T.; Ranasinghe, D.; Zakrzewski, V. G.; Gao, J.; Rega, N.; Zheng, G.; Liang, W.; Hada, M.; Ehara, M.; Toyota, K.; Fukuda, R.; Hasegawa, J.; Ishida, M.; Nakajima, T.; Honda, Y.; Kitao, O.; Nakai, H.; Vreven, T.; Throssell, K.; Montgomery, J. A., Jr.; Peralta, J. E.; Ogliaro, F.; Bearpark, M. J.; Heyd, J. J.; Brothers, E. N.; Kudin, K. N.; Staroverov, V. N.; Keith, T. A.; Kobayashi, R.; Normand, J.; Raghavachari, K.; Rendell, A. P.; Burant, J. C.; Iyengar, S. S.; Tomasi, J.; Cossi, M.; Millam, J. M.; Klene, M.; Adamo, C.; Cammi, R.; Ochterski, J. W.; Martin, R. L.; Morokuma, K.; Farkas, O.; Foresman, J. B.; Fox, D. J. Gaussian, Inc., Wallingford CT, 2016.

14 Zhao, Y. & Truhlar, D. G. The M06 suite of density functionals for main group thermochemistry, thermochemical kinetics, noncovalent interactions, excited states, and transition elements: two new functionals and systematic testing of four M06-class functionals and 12 other functionals. *Theoretical Chemistry Accounts* **120**, 215-241 (2008).

15 Ermanis, K., Parkes, K., Agback, T. & Goodman, J. Doubling the power of DP4 for computational structure elucidation. *Organic & biomolecular chemistry* **15**, 8998-9007 (2017).

16 Lodewyk, M. W., Siebert, M. R. & Tantillo, D. J. Computational prediction of 1H and 13C chemical shifts: A useful tool for natural product, mechanistic, and synthetic organic chemistry. *Chemical Reviews* **112**, 1839-1862 (2011).
17 CHESHIRE, CHEmical SHift REpository with Coupling Constants Added Too; http://cheshirenmr.info.
18 Steinbeck, C., Krause, S. & Kuhn, S. NMRShiftDBConstructing a Free Chemical Information System with Open-Source Components. *Journal of Chemical Information and Computer Sciences* **43**, 1733-1739, doi:10.1021/ci0341363 (2003).
19 Arnó, M., González, M. A. & Zaragozá, R. J. Synthesis of C-17-Functionalized Spongiane Diterpenes: Diastereoselective Synthesis of (−)-Spongian-16-oxo-17-al,(−)-Acetyldendrillol-1, and (−)-Aplyroseol-14. *The Journal of organic chemistry* **68**, 1242-1251 (2003).
20 Rychnovsky, S. D. Predicting NMR spectra by computational methods: Structure revision of hexacyclinol. *Organic letters* **8**, 2895-2898 (2006).
21 Johnson, W. M., Littler, S. W. & Strauss, C. R. Structural revision and synthesis of sinharine and methylsinharine. *Australian Journal of Chemistry* **47**, 751-756 (1994).
22 Chmurny, G. N. *et al.* 1H-and 13C-NMR assignments for taxol, 7-epi-taxol, and cephalomannine. *Journal of natural products* **55**, 414-423 (1992).
23 Saielli, G., Nicolaou, K., Ortiz, A., Zhang, H. & Bagno, A. Addressing the stereochemistry of complex organic molecules by density functional theory-NMR: Vannusal B in retrospective. *Journal of the American Chemical Society* **133**, 6072-6077 (2011).
24 Barone, G. *et al.* Determination of the relative stereochemistry of flexible organic compounds by ab initio methods: conformational analysis and Boltzmann‐averaged GIAO 13C NMR chemical shifts. *Chemistry–A European Journal* **8**, 3240-3245 (2002).
